# Sparse Representation-Based Denoising for High-Resolution Brain Activation and Functional Connectivity Modeling: A Task fMRI Study

**DOI:** 10.1109/access.2020.2971261

**Published:** 2020-02-03

**Authors:** SEONGAH JEONG, XIANG LI, JIARUI YANG, QUANZHENG LI, VAHID TAROKH

**Affiliations:** 1 School of Electronics Engineering, Kyungpook National University, Daegu 14566, South Korea; 2 Department of Radiology, Massachusetts General Hospital and Harvard Medical School, Boston, MA 02114, USA; 3 Biomedical Engineering, Boston University, Boston, MA 02215, USA; 4 Rhodes Information Initiative at Duke, Durham, NC 27708, USA

**Keywords:** Task fMRI, fMRI denoising, dictionary learning and sparse coding

## Abstract

In the field of neuroimaging and cognitive neuroscience, functional Magnetic Resonance Imaging (fMRI) has been widely used to study the functional localization and connectivity of the brain. However, the inherently low signal-to-noise ratio (SNR) of the fMRI signals greatly limits the accuracy and resolution of current studies. In addressing this fundamental challenge in fMRI analytics, in this work we develop and implement a denoising method for task fMRI (tfMRI) data in order to delineate the high-resolution spatial pattern of the brain activation and functional connectivity via dictionary learning and sparse coding (DLSC). In addition to the traditional unsupervised dictionary learning model which has shown success in image denoising, we further utilize the prior knowledge of task paradigm to learn a dictionary consisting of both data-driven and model-driven terms for a more stable sparse representation of the data. The proposed method is applied to preprocess the motor tfMRI dataset from Human Connectome Project (HCP) for the purpose of brain activation detection and functional connectivity estimation. Comparison between the results from original and denoised fMRI data shows that the disruptive brain activation and functional connectivity patterns can be recovered, and the prominence of such patterns is improved through denoising. The proposed method is then compared with the temporal non-local means (tNLM)-based denoising method and shows consistently superior performance in various experimental settings. The promising results show that the proposed DLSC-based fMRI denoising method can effectively reduce the noise level of the fMRI signals and increase the interpretability of the inferred results, therefore constituting a crucial part of the preprocessing pipeline and provide the foundation for further high-resolution functional analysis.

## I. INTRODUCTION

In neuroimaging field, functional Magnetic Resonance Imaging (fMRI) is widely used to localize the task-evoked brain activation and to delineate the temporal and spatial correlation patterns of functional connectivity as well as to perform the early diagnosis of various brain disorders [1]–[7]. However, the inherently low signal-to-noise ratio (SNR) of fMRI signals due to the low signal change of BOLD recording, plus various types of modality-specific noise and artifacts limits the reliability, accuracy, as well as reproducibility of the fMRI-based analysis and applications. The presence of noise in the fMRI data makes it difficult to identify the subtle differences in activation and functional connectivity, and therefore leads to the low sensitivity of the statistical inferences. In order to reduce the noise and enhance the SNR, fMRI images are typically preprocessed prior to the analysis via filtering [1]–[3], [7]–[10]. Specifically, in [2], the fMRI data are further preprocessed by low-pass temporal filtering, head-motion regression, whole brain signal regression, and ventricular and white matter signal regression after compensating the T1-equilibration effects, slice acquisition-dependent time shifts via SPM2 [11] and head motion via FMRIB Software Library (FSL) [12]. The fMRI data in [9] are temporally filtered using a high pass filter to maximize alignment across image modalities, to minimize distortions relative to the subject’s anatomical space and to minimize spatial smoothing (blurring) of the data. [10] proposes a general wavelet-based denoising scheme for fMRI data by using Gaussian-based filter that is verified to introduce the less smoothing preserve the sharpness of the images and retain the original shapes of active regions. The temporal non-local means (tNLM) filtering [8] has been shown to be able to reduce the local fluctuations without too much spatial blurring by exploiting the temporal information for the weighting.

With the advances in MRI imaging techniques for the high-resolution acquisitions, more robust and effective denoising methods beyond image filtering are needed for more precisely pinpointing the analytics results. To address the demands for denoising the neuroimaging data, motivated by the recent advances of denoising in the natural image processing field [13], in this work we propose a dictionary learning and sparse coding-based task fMRI (tfMRI) denoising method to enable the high-resolution brain activation and functional connectivity analysis. The dictionary learning and sparse coding (DLSC) methodology introduced in machine learning and pattern recognition fields [14], [15] has been applied for natural image denoising [13]. In addition, a variety of the fMRI studies are motivated by the finding of the sparse response principle of the neural activity in brain [16], [17] which facilitates the development of DLSC-based techniques for fMRI data [18]–[23]. The basic idea of the DLSC-based fMRI analysis is to learn a set of the underlying hemodynamic signals and represent the original huge data matrix aggregating all the fMRI signals within the whole brain of one subject as a sparsity-regularized linear combination of the learned basis signals. In general, the DLSC-based methods are data-driven which can be efficient in learning adaptive and over-complete representations [14], [15]. Moreover, it has been reported in the literatures [18], [20], [23]–[25] that the performance of DLSC-based methods can be further improved by utilizing the prior knowledge in a supervised way.

In our proposed method, in order to overcome the limitations of the unsupervised DLSC techniques such as absence of the interpretation of the model output and lack of statistical power [9], [21], [26], [27], we utilize the prior knowledge of task paradigm during the learning steps to train the model-driven and data-driven dictionaries separately and model their corresponding sparse representations, yielding an enhanced denoising performance. Specifically, we consider two different sets of dictionary basis functions: fixed atoms and learned atoms. The fixed atoms are predefined as the task stimulus curves which are generated by the convolution of a canonical hemodynamic response function (HRF) and the simple boxcar stimulus function indicating each occurrence of a generation event. On the other hand, the learned atoms are trained in an unsupervised approach from the reduced signal matrix which is only a portion of the original signal matrix. The reduced signal matrix is constructed by selecting fMRI signals from the original data which are not correlated with the task stimulus, based on the rationale that signals correlated with the tasks can be represented and reconstructed by the fixed atoms. Using a reduced input for the training is a unique feature of our proposed method and makes it different from the existing supervised/semi-supervised DLSC-based techniques [18], [20], [23]–[25], and it has been shown in the results that such feature can resolve the challenges caused by the enormous size of voxel-wise fMRI data as well as the collinearity among signals. The final dictionary consisting of the fixed and learned atoms is then used to sparsely code the input fMRI signals on each voxel for denoising, as the noise patterns in the result signals will be partially removed during the sparse coding process [24], [25].

For model validation and performance evaluation, we apply the proposed DLSC-based denoising method to both the synthetic dataset and the Human Connectome Project (HCP) motor tfMRI dataset [28]. It is observed from the results that the proposed denoising method can conserve and strengthen the meaningful activation pattern, while recovering the missing activation (false negative activation detection results from analytics such as GLM) disrupted by noise from the fMRI signals. Furthermore, functional connectivity analysis on the denoised data shows pronounced effects on the connectivity strengths, revealing consistent and neuroscientifically meaningful high-resolution connectivity patterns in the brain.

## II. MATERIALS AND METHODS

### A. DATA ACQUISITION AND PREPROCESSING

In this work, we focus on the Motor task, while the proposed method can be easily adopted to and have been validated in other types of tfMRI data as well, including (but not limited to) Working Memory, Gambling, Language, Social Cognition, Relational Processing and Emotion processing within the HCP database. The HCP tfMRI data is acquired with the following parameters: TR = 720ms, TE = 33. 1ms, flip angle = 52°, BW = 2290Hz/Px, in-plane FOV = 208 × 180mm, 72 slices and 2.0mm isotropic voxels. During the experiments, the participants are informed with the visual cues to tap their left or right fingers, squeeze their left or right toes and move their tongues. These movements are verified to identify the effector corresponding to the specific activation individually in motor areas [2], [7]. Each run consists of 13 blocks with 2 left hand (LH) and 2 right hand (RH) movements, 2 left foot (LF) and 2 right foot (RF) movements, 2 tongue (T) movements and three 15s fixation blocks. Each block is 12s and preceded by the 3s visual cue. The number of frames per run for each subject is 284 and the total run duration is 214s. The HCP minimally preprocessing pipeline [28] includes motion correction, slice time correction, spatial smoothing, high-pass filtering and non-linear (FNIRT) registration [29] into MNI152 space. For each subject, the fMRI signals are extracted on each gray matter voxel and arranged into a single 2D matrix. The details for data acquisition and experiment design can be found in [28].

### B. DICTIONARY LEARNING AND SPARSE CODING (DLSC)-BASED DENOISING

The DLSC technique is an unsupervised learning algorithm developed in machine learning and pattern recognition fields [14], [15]. By learning a set of basis vectors with the sparsity constraint and representing the original signals as the linear combinations of the learned bases, the DLSC approach can reconstruct the audio, image and video data. In addition, it has been reported that incorporating the prior information into the basis vector estimation can provide further improvements of the DLSC-based methods in a supervised or semi-supervised way, which has been applied to the fMRI analysis as well [18], [20], [23]–[25].

**FIGURE 1.**
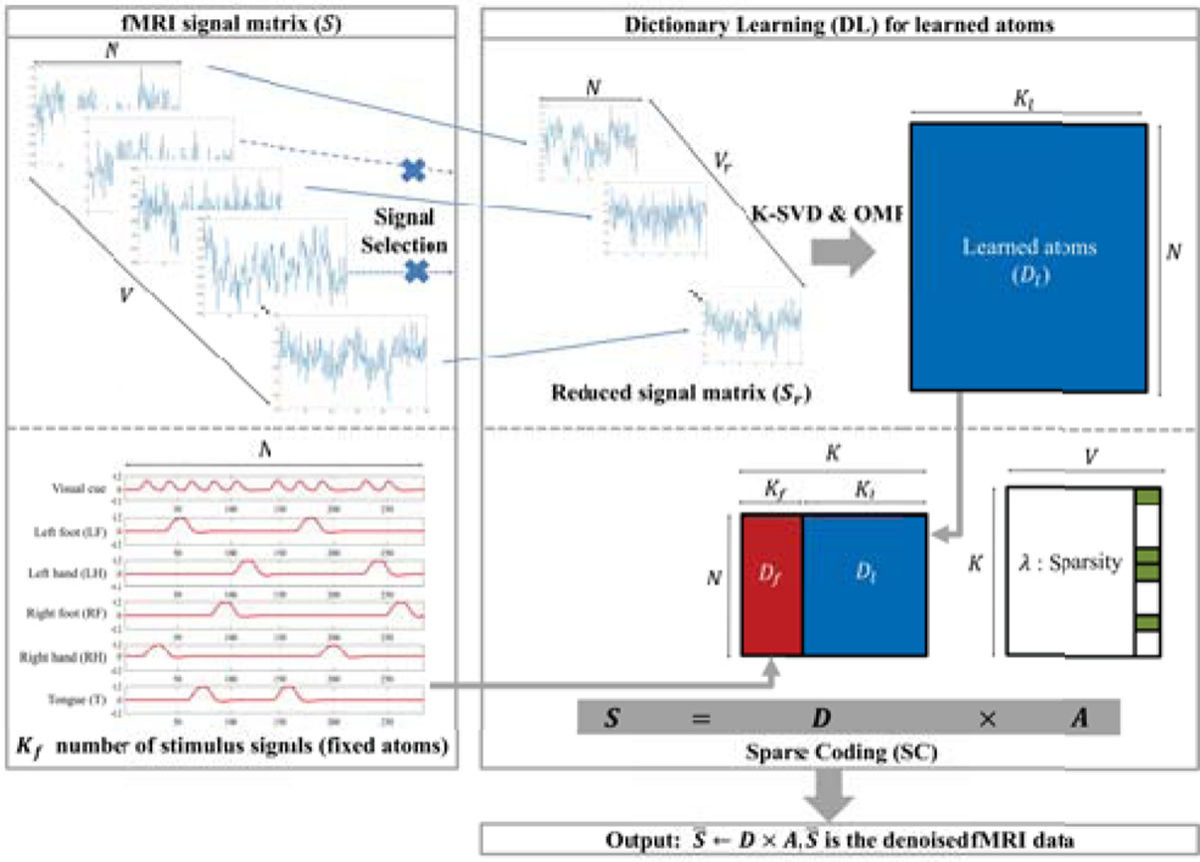
The algorithmic pipeline of the proposed DLSC-based denoising method. There are two different types of dictionary bases: fixed atoms and learned atoms. These two types of atoms construct the sub-dictionary matrices *D*_*f*_ and *D*_*l*_, which lead to the corresponding sub-coefficient weighting matrices *A*_*f*_ and *A*_*l*_, respectively.

In this work, we propose a DLSC-based denoising method as illustrated in Fig. 1. The basic principle is to utilize the two different types of dictionary atoms such as i) fixed atoms and ii) learned atoms. The fixed atoms are predefined as the task stimulus curves. The curves are generated by convoluting a SPM [11] canonical HRF with the boxcar stimulus function, as shown in Supplemental Fig. 1. The canonical HRF is the basis of the parametric model that can estimate the changes in the fMRI blood oxygen level dependent (BOLD) signal evoked by an instantaneous burst of activation. Accordingly, the number of fixed atoms is equal to the number of stimuli in the task of interest. We consider the six different stimulus curves for the visual cues and the mentioned five movements, namely LF, LH, RF, RH, T, according to the HCP Motor task paradigm. These curves will also be applied to construct the regressors for the general linear model (GLM) analysis to find the activation maps of the particular movements whose details will be discussed in Section III-A. The use of HRF-convoluted task paradigm during the learning has two motivations. Firstly, the stimulus curves are designed to be used for making more clear inferences about regional brain activities and connectivities in functional neuroimaging, which is also the goal of our denoising method. In addition, from an algorithmic perspective, by incorporating the fixed atoms, we can improve the effectiveness of the learning procedure and avoid converging to the local minimum. Furthermore, the fact that a large portion of variation in the tfMRI signal is related to the stimulus will inevitably cause the intra-correlation among atoms which is a major malfunction factor for the learning, while the correlation constraint in the proposed method can overcome this difficulty.

Specifically, for each subject, the entire brain fMRI signals are extracted and the signal at each voxel is normalized to zero-mean and unit variance, yielding the *N* × *V* fMRI signal matrix ***S***, where *N* is the number of time samples or frames and *V* is the number of voxels. By using the proposed DLSC-based denoising method, the signal matrix ***S*** can be factorized into:

(1) S=D×A,

where ***D*** is the *N* × *K* dictionary matrix consisting of the *K* atoms {*d*_*k*_} with *k* = 1, . . . , *K* of the underlying hemodynamic signals and ***A*** is the corresponding *K* × *V* coefficient matrix to collect the sparse representations {***a***_*v*_} with *v* = 1, . . . , *V* for fMRI signal. In (1), the dictionary matrix *D* is defined as ***D*** = [***D***_*f*_, ***D***_*l*_]. ***D***_*f*_ is the *N* × *K*_*f*_ sub-dictionary matrix consisting of the *K*_*f*_ fixed atoms which are the stimulus curves related to the task design (Supplemental Fig. 1). ***D***_*l*_ is the *N* ×*K*_*l*_ sub-dictionary matrix consisting of the *K*_*l*_ learned atoms, satisfying the condition of *K* = *K*_*f*_ + *K*_*l*_.

In order to train the learned dictionary ***D***_*l*_, we construct the *N* × *V*_*r*_ signal matrix ***S***_*r*_, which is a subset of ***S*** and only includes fMRI signals whose correlation value with the fixed atoms is less than a predefined threshold *C*_*th*_: ]

(2a) $$S_{r}=\{\left(s_{i}\right)\in {\mathbb{R}}^{N\times V_{r}}|\mathrm{corr}\left(s_{i},d_{k}\right)\leq C_{th},\;\;i=1,\ldots ,V,\;k=1,\ldots ,K_{f}\},$$

where corr(·) represents the Pearson correlation operation. With the sub-matrix ***S***_*r*_, we then train the learned dictionary ***D***_*l*_ by solving the following l-0 regularized minimization problem:

(3a) $$\underset{D_{l},A^{*}}{\mathrm{minimize}}{\left\|S_{r}-D_{l}A^{*}\right\|}_{F}^{2}$$

(3b) $$\text{s}.\text{t}.,\;{\left\|a_{v}^{*}\right\|}_{0}\leq \lambda \;v=1,\ldots ,V,$$

where ∥ · ∥_*F*_ and ∥ · ∥_0_ indicate the Frobenius norm and the zero norm (i.e. counting the non-zero elements in the matrix), respectively. A* is the *K*_*l*_×*V*_*r*_ coefficient matrix composed of the sparse coding ${\left\{a_{v}^{*}\right\}}_{v=1,\ldots ,V_{r}}$ of the *V*_*r*_ voxels in the matrix ***S***_*r*_, and *λ* represents the sparsity constraint on the maximum number of non-zero coefficients for the signal at each voxel. The threshold *C*_*th*_ is selected to satisfy the condition that the number of voxels in reconstructed matrix ***S***_*r*_ is larger or equal to the number of learned atoms, i.e. *V*_*r*_ ≥ *K*_*l*_. The use of the submatrix ***S***_*r*_ of the original signals for the learning can mitigate the potential malfunction caused by the intra-correlation between fixed atoms and learned atoms. It can resolve the difficulties in processing the large-scale fMRI data by excluding the substantial number of voxels from the original signal matrix. The analytic procedure of splitting the data and the dictionary into the fixed and learned parts, then using the submatrix Sr for the dictionary learning is the main innovation of our proposed method. Comparing with previous DLSC-based fMRI studies [20], [23], this semi-supervised procedure enables more accurate analysis as well as reduces the computational cost for the learning. In order to solve the optimization problem in (3), K-SVD algorithm [13] and Orthogonal Matching Pursuit (OMP) [30] are adopted, where K-SVD algorithm coupled with OMP updates the learned dictionary ***D***_*l*_ in an iterative and alternative fashion with the sparse coding A*. Subsequently, the coefficient matrix ***A*** = [***A***_*f*_; ***A***_*l*_] is calculated by using OMP based on the designed dictionary matrix ***D*** consisting of the fixed dictionary *D*_*f*_ and the learned dictionary ***D***_*l*_, where ***A***_*f*_ is the *K*_*f*_ × *V* coefficient matrix related to the task-driven network and ***A***_*l*_ is the *K*_*l*_ × *V* coefficient matrix related to the intrinsic network, e.g., resting-state network. Finally, we denoise the signal matrix *S* which is denoted as $\bar{S}\leftarrow D\times A$.

### C. PARAMETER TUNING

When running the DLSC-based denoising method, the parameters of the dictionary size *K*, the sparsity *λ* and the threshold *C*_*th*_ for constructing the matrix ***S***_*r*_ need to be tuned. However, there is no theoretical criterion for determining the parameters *K* and *λ* for the dictionary learning analysis in neuroscience studies [31]. And for the parameter *C*_*th*_, the correlation strength of the fMRI signals with the stimulus varies across different tasks, thus it is difficult to find a universal standard for a good threshold for separating task-related and task-free voxels. Therefore, we perform a grid-search for finding the best parameter combinations with *K* = 300 to 500 (step size 100), *λ* = 5 to 50 (step size 5) and *C*_*th*_ = 0.1 to 0.4 (step size 0.1). Finally we choose (*K, λ,C*_*th*_) = (400, 40, 0.1) based on visual inspection of the GLM results where denoised signals are used as input, as well as the empirical parameters reported previously [23], [31], [32]. The GLM activation maps with the different parameter combinations during the grid-search can be found in Supplemental Fig. 2, Fig. 3 and Fig. 4. The dictionary size *K* of 400 is in accordance with the theoretical finding in [14] that the learned dictionary shall be over-complete. In our case, the dictionary size should be larger than the number of frames of fMRI image (284). For the threshold value *C*_*th*_, it is observed in the grid-search experiment that the GLM results are not significantly affected by the correlation threshold, while the computational time for denoising rapidly decreases as a low *C*_*th*_ reduce the size of the submatrix ***S***_*r*_. On the other hand, for dictionary learning it is required that *V*_*r*_ ≥ *K*_*l*_ (i.e. number of samples in the input matrix is larger than the dictionary size) to avoid redundancy of the learned dictionaries. Therefore, we set the threshold *C*_*th*_ as its minimum possible value of 0.1 in this work.

## III. RESULTS

In this work, the proposed DLSC-based denoising method is applied on the synthetic data generated by the Gaussian noise-based simulation, as well as the HCP motor tfMRI dataset. In both experiments, the denoising performances are evaluated based on the results from functional localization analysis (activation detection) and functional connectivity analysis. For comparison, we also adopt the temporal non-local means (tNLM) [8] method, which has been widely used to effectively reduce the local fluctuations to obscure larger scale behavior, for denoising the same set of fMRI data. Following [8], we set the distance parameter of tNLM as 11 and the smoothing level as 0.72 that empirically provides the best result on the HCP Motor tfMRI dataset. The GLM analysis is performed using SPM12 software [11], with six event-related designs (visual cue, LF, LH, RF, RH, T) for HCP Motor task and their stimulus paradigms convolved with the canonical HRF basis function. Except for the infinite masking threshold and high-pass filtering with a cutoff period set to be the maximum interval of the stimulus repetition, the default SPM settings are used in all processing steps, e.g., global AR(1) auto correlation correction, Microtime resolution 16 and Microtime onset 8. The t-statistic is applied by setting the p-value to 0.001 (uncorrected for multiple comparison test), and setting the zero-spatial extent threshold voxel.

### A. MODEL VALIDATION AND PERFORMANCE COMPARISON ON SYNTHETIC DATA (REAL DATA WITH MANUALLY CONTROLLED ADDED NOISE)

In this work, we validate the effectiveness of the proposed model based on its denoising performance on synthetic data where various levels of Gaussian noise are added to the real fMRI dataset. Specifically, synthetic fMRI signals are generated by contaminating one HCP Motor tfMRI data from a randomly-selected subject with additive zero-mean Gaussian noise *N*(0, *σ*^2^). Various standard deviations (*σ* = 100, 200 and 300) are tested in this study, resulting in synthetic data with average Signal-to-Noise Ratio (SNR) of 38.26dB, 32.21dB and 28.69dB respectively. The effectiveness of denoising is evaluated based on the brain functional activation detection results of the GLM [33], [34] with SPM12 software [11]. The basic rationale of the synthetic data experiment is that the Gaussian noises in the contaminated signals will hinder the regression-based GLM analysis and degrade the results (which has also been a challenge in practical fMRI applications), while the denoising method is supposed to recover the original signals without introducing any bias into the data.

**FIGURE 2.**
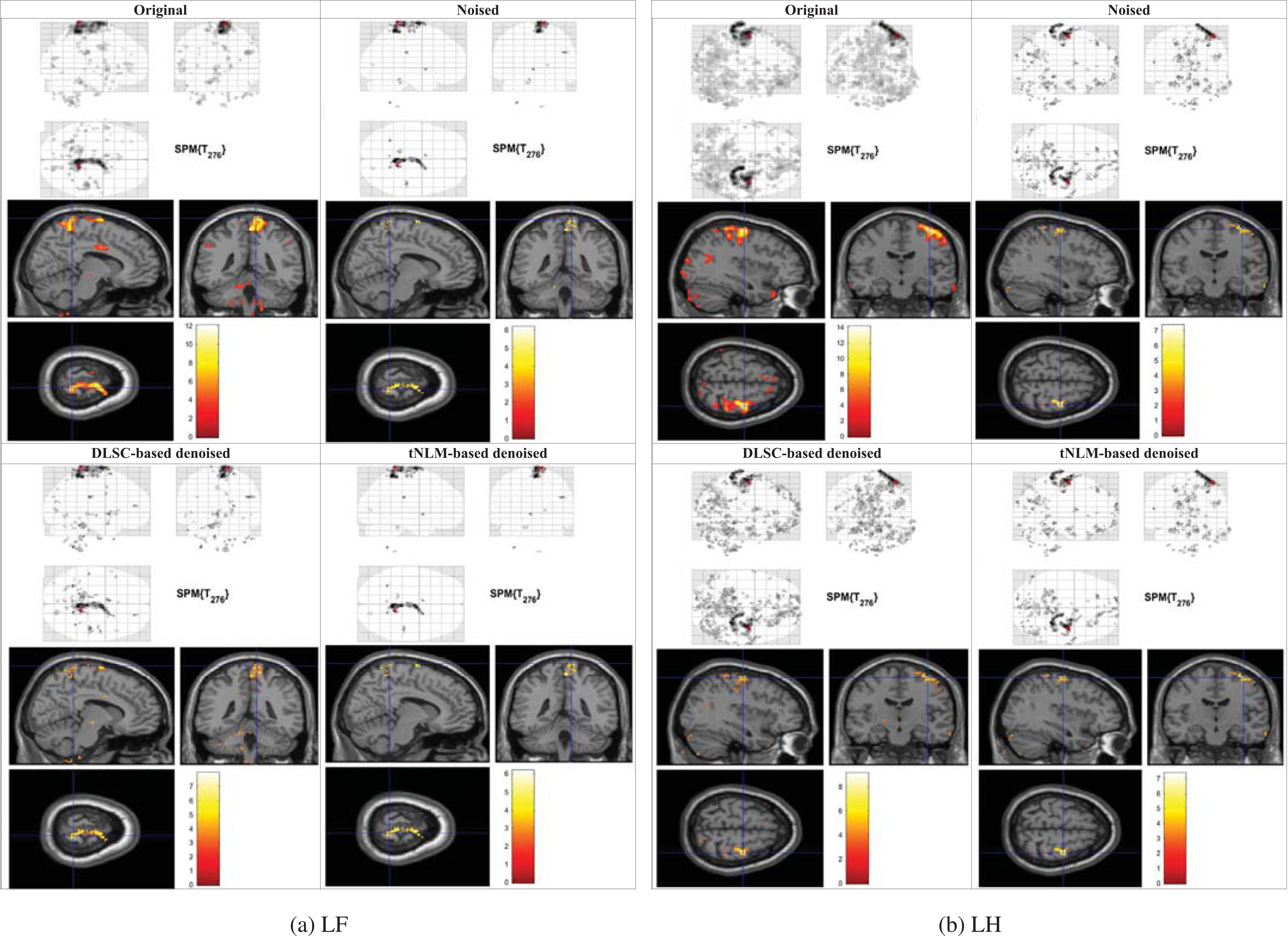
GLM-derived activation maps of the original, synthetic (additive Gaussian noise with *σ* D 200) and denoised tfMRI data by DLSC-based / tNLM-based methods for the LF and LH movements in HCP Motor tfMRI data.

The GLM activation detection results of the original, synthetic (additive Gaussian noise with *σ* = 200) and denoised fMRI data (by both the proposed DLSC-based method and the tNLM-based method) of two sample movement types (Left Foot/LF and Left Hand/LH) are illustrated in Fig. 2. For each set of results, the glass brain visualization on the upper panel shows the activated voxels with t-value exceeding the threshold (corresponding to p-value = 0.001). The bottom panel shows the activation map of the locations with the maximal t-value overlaid on the T-1 weighted anatomical image, where the color bar indicates the corresponding t-value. Result visualizations with various noise levels from all the five movements (LF, LH, RF, RH and T) in the HCP Motor tfMRI data can be found in Supplemental Fig. 5 and Fig. 6.

In this experiment, the activation maps obtained by the GLM analysis on the original, high-quality fMRI data are regarded as ground truth. From Fig. 2 it can be found that the activation maps estimated from the denoised data using DLSC are more consistent with the ground truth, comparing with the results from the noised data as well as the tNLM-based denoised data. The results indicate that the original activation maps ruined by the additive Gaussian noise can be recovered by the proposed DLSC-based denoising method. Moreover, most of the recovered activation detection results after denoising by DLSC can be found in the ground truth, showing that the preprocessing step is not introducing any observable bias towards the GLM-based analysis.

In order to quantitatively compare the model performance, for each movement type we analyze the spatial similarity between pairs of activation maps estimated from the noised/denoised signals and the activation maps estimated from the ground truth (original) signals. The spatial similarity is measured in terms of Dice Similarity Coefficient (DSC), defined as:

(4) $$\text{DSC}=\frac{\left|X\cap Y\right|}{\left|X|+|Y\right|},$$

where | · | counts the number of voxels in one voxel set (activation map), *X* is the set of voxels with *t* ≥ *T*_*th*_ (*T*_*th*_ = 3.12) in the ground truth results estimated from the original fMRI data, while *Y* is the set of *t* ≥ *T*_*th*_ voxels in the results estimated from noised/denoised data. For example, the notation DSC_*DLSC*_ measures the similarity between the activation map estimated from the proposed DLSC-based denoised data and that from the original ground truth data. We further calculate the ratio between corresponding DSCs to quantify the improvements of the activation detection results by the denoising methods:

(5a) $$r_{DLSC/\;Noised\;}=\frac{{\text{ DSC}}_{DLSC}}{{\text{ DSC}}_{Noised\;}}\times 100(\%),$$

(5b) $$r_{tNLM/Noised\;}=\frac{{\text{ DSC}}_{tNLM}}{{\text{ DSC}}_{Noised\;}}\times 100(\%)$$

For example, *r*_*DLSC/Noised*_ shows the improvement of the DLSC-denoised results over the noised results. The similarity measurements and the improvement ratios are summarized in Table. We also test the denoising effect on the original signals without noise (rows with *σ* = 0 in Table 1, in order to investigate whether the proposed method can faithfully reconstruct the data.

The similarity measurements and the corresponding improvement ratios show that the DLSC-based denoising method can help recovering the result activation maps towards the ground truth, where its effectiveness increases with noise level as seen in the *r*_*DLSC/Noised*_ column. On the contrary, the tNLM-based denoising decreases the similarity of the estimated activation maps with the ground truth at low noise levels (*σ* = 100 and *σ* = 200). This is mainly caused by the fact that tNLM is essentially based on signal averaging (either spatially across voxels or temporally across volumes), thus it suffers from the effect of smoothing (or over-smoothing in certain circumstances) when performing voxel-wise estimation such as calculating DSC.

### B. FUNCTIONAL LOCALIZATION: INDIVIDUAL-LEVEL AND GROUP-LEVEL ANALYSIS ON HCP DATA

**FIGURE 3.**
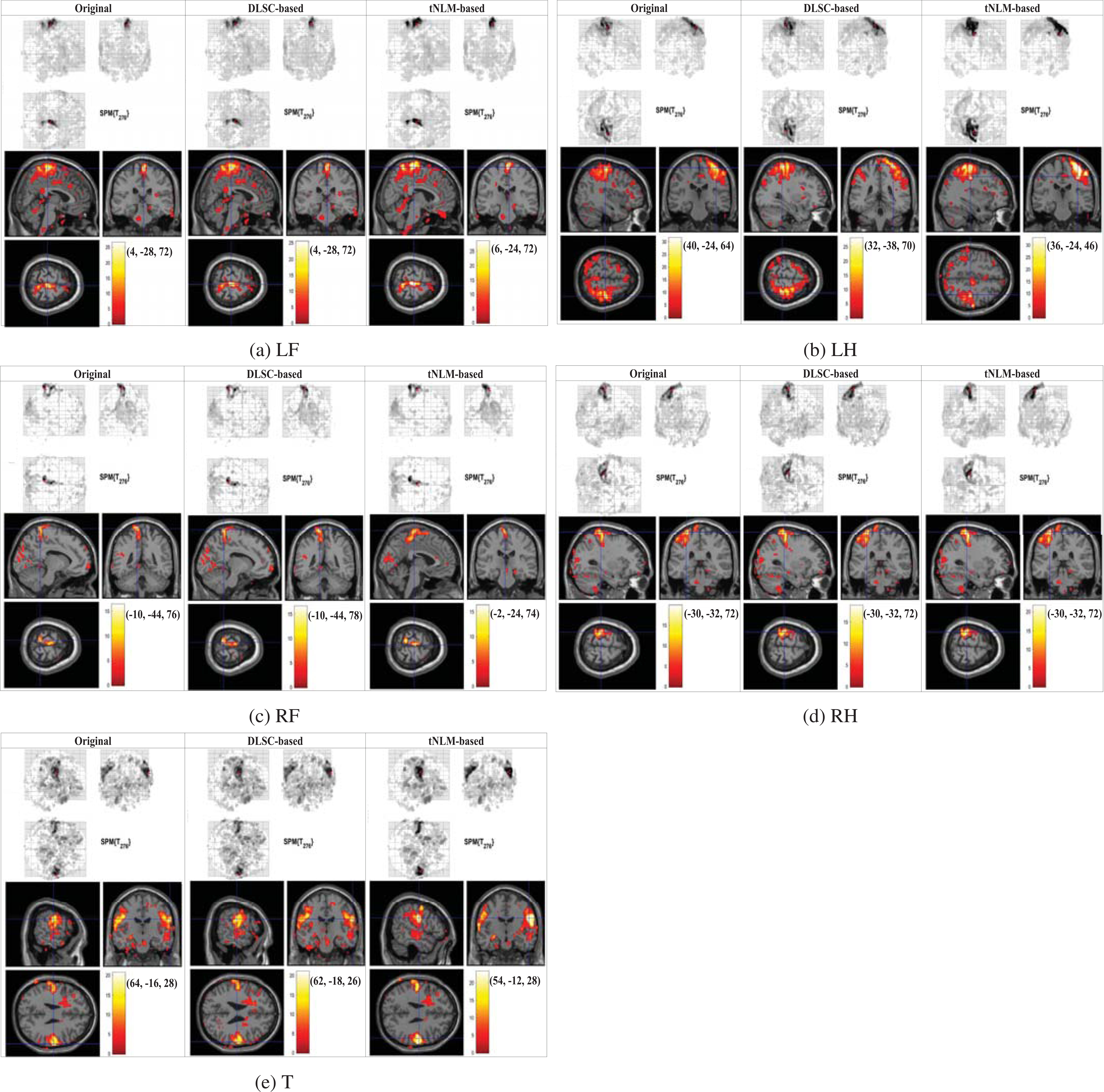
Individual-level GLM-derived activation maps of a randomly-selected subject obtained from the original Motor tfMRI data (left), DLSC-based denoised data (middle) and tNLM-based denoised data (right). Results of the five movement types ((a) LF, (b) LH, (c) RF, (d) RH and (e) T) are listed in the five rows respectively.

In this section the performance of the denoising methods (DLSC-based and tNLM-based) are evaluated on the real data from all the 68 subjects of the HCP Q1 Motor tfMRI database. Following a similar approach as used in section III-A, we apply GLM with SPM12 software [11] on the real tfMRI data for estimating both the individual-wise and group-wise activation maps, where the first and second-level GLM analysis in SPM are used respectively. First, we compare the individual-level GLM-based activation detection results for the five movement types (LF, LH, RF, RH and T) obtained from the raw data, DLSC-based denoised data and tNLM-based denoised data. The GLM results of one randomly-selected subject for five movement types are shown in Fig. 3, where the locations of maximal activations, the heatmap of the T-values and the MNI coordinates of the maximal activations are provided respectively. Since there is no functional lateralization considered for the tongue movement, the maximal activated regions can be located in left and/or right hemisphere. A complete list of individual-level GLM results from all the 68 subjects can be found in Supplemental Materials.

**FIGURE 4.**
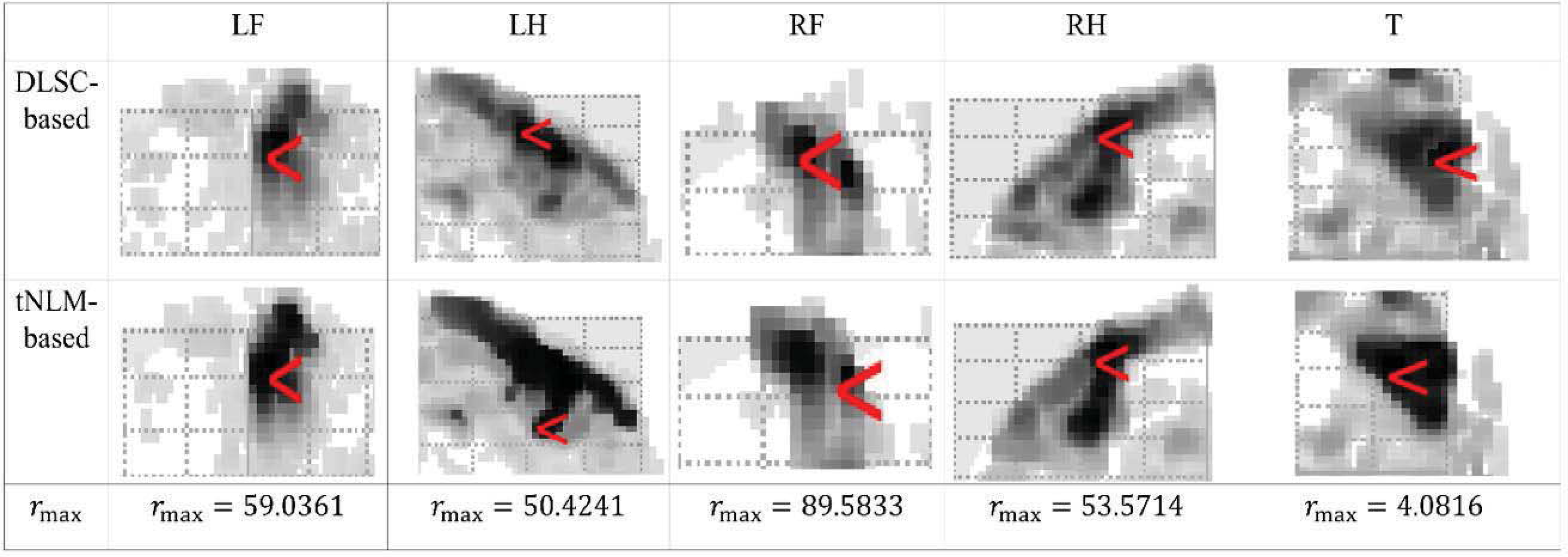
Zoom-in of the maximal activation in the individual-level GLM-derived activation maps of a randomly-selected subject after denoising by DLSC-based and tNLM-based methods for LF, LH, RF, RH and T movement types. The red arrow points to the voxel with maximal activation. The ratio *r*_max_ is defined in Eq. (6).

From Fig. 3, it can be found that the GLM results from data denoised by both our proposed method and the tNLM-based method are improved in correspondence with the GLM results from the original data and correct maximal activation can be estimated, showing that denoising process is not introducing any bias into the signals. Further, both methods can lead to larger area of activated regions for the detection than the results from original data, showing that denoising is effectively increasing the signal quality and interpretability of the results. In addition, comparing with tNLM, less spatial blurring (smoothing) effect could be observed from the results of the proposed DLSC-based denoising method. Specifically, the boundaries of activated regions in GLM results from the tNLM-denoised signals tend to be blurred, resulting in large and rough clusters for certain datasets. While our proposed DLSC-based method allows sharper and more concentrated activation regions in the GLM results as it utilizes inherent sparsity of the images as prior for denoising. To better illustrate the subtle differences of the results between the two methods, we magnify the cluster with the maximal activations from DLSC-based and tNLM-based denoised data in Fig. 4 below. For the quantitative comparison of these resulting clusters with maximal activations, we calculate the ratio *r*_max_ between the numbers of voxels in the resulting clusters estimated from DLSC-based denoised data (|*X*_*DLSC*_|) and tNLM-based denoised data (|*X*_*tNLM*_|), indicating the size difference between the resulting clusters derived from the two methods. The cluster is defined by the voxels with normalized t-value larger than the pre-defined threshold *T*_*th*_ (set as 12 in this work):

(6) $$r_{\max}=\frac{\left|X_{DLSC}\right|}{\left|X_{tNLM}\right|}\times 100(\%).$$

From Fig. 4, it can be observed that, although the overall location and spatial pattern of the resulting cluster with the maximal activation derived from the two methods are similar, the tNLM-based denoising will lead to much larger clusters comparing with the proposed DLSC-based method. The measurement *r*_max_ further shows that the size of the cluster estimated from the data denoised by DLSC is around 50% smaller than tNLM while maintaining the accurate spatial pattern for the results, thus the precision of the results is effectively doubled. Considering that the size of each voxel (2mm isotropic) is already quite large in terms of the number of neuronal cell types it can cover, correctly pinpointing the activation regions through denoising by the proposed method will be a fundamental feature for high-resolution neuroscience studies.

**FIGURE 5.**
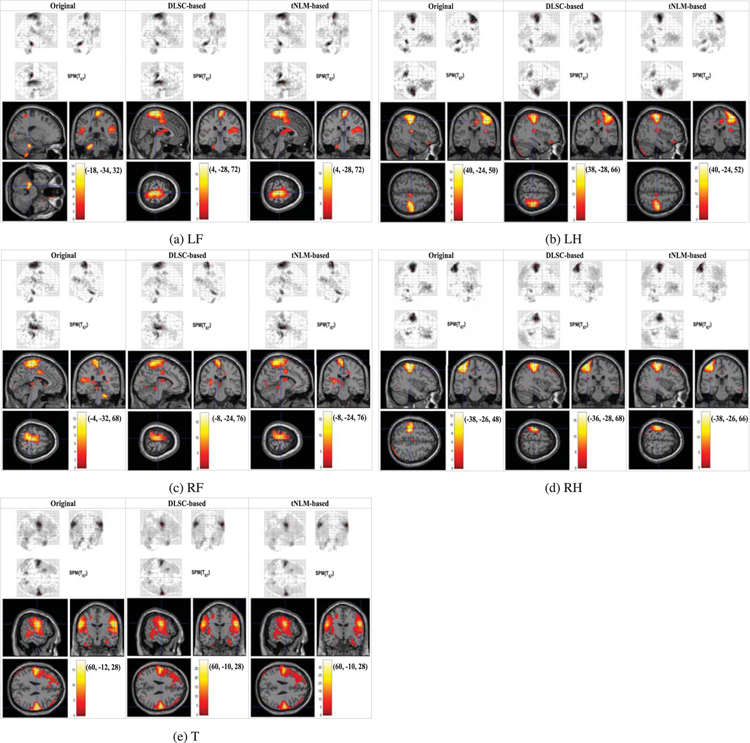
Group-level GLM-derived activation maps of the original data, DLSC-based denoised data and tNLM-based denoised data, for (a) LF, (b) LH, (c) RF, (d) RH and (e) T movement types.

In addition to the individual-level GLM-based activation detection results, we also obtain the group-level GLM activation maps from 68 subjects, as shown in Fig. 5. Comparison between the denoised results (both by DLSC and tNLM) and the original results shows that clearer pattern of activation map can be obtained after denoising, and the two denoising methods are generating consistent results. Specially, the group-level GLM activation maps show high deviation between the results estimated from LF and RF movements of the original data. Such asymmetry of the activation detection is then corrected by both DLSC-based and tNLM-based methods after denoising.

In order to provide a ground truth for the evaluation and quantitative comparison for the model performance, we extract the regions of interest (ROIs) from the parcellation studies as introduced in [9] which is also performed on the HCP Q1 dataset. As the parcellation framework utilizes multiple modalities of images from the HCP database (MRI, rsfMRI and tfMRI) and is supposed to establish the fingerprint for precise and consistent region definition, these ROIs are then considered as ground truth for evaluating our activation detection results. The similarities between the ground truth parcellation ROIs and the activation maps estimated from the three different tfMRI data (original, DLSC-denoised, tNLM-denoised) are then calculated in terms of DSC as defined in Eq. (4), where the ground truth image ***X*** is the parcellation ROI and ***Y*** is the spmT image obtained from the group-level GLM analysis on the corresponding fMRI data (original/denoised). Specifically, the outlines of ROIs are extracted from the HCP sub-area definition provided in [9] for each movement type, and then mapped into the volumetric space with the depth of 2mm. Note that since the parcellation in the left and right hemispheres for tongue movement are separately given in the parcellation, we use the union set of these ROIs for the comparison. The similarity measurements in DSC are summarized in Table 2. Furthermore, in order to provide a more intuitive comparison between the improvement on DSC (i.e. similarity with the ground truth) achieved by the DLSC-based and tNLM-based denoising methods, we define the improvement ratios *r*_*DLSC/Ori*_ and *r*_*tNLM*_*/*_*Ori*_ following the similar concept in Eq. (5a) and Eq. (5b), which are also listed in Table 2:

(7a) $$r_{DLSC/Ori}=\frac{{\mathrm{DSC}}_{DLSC}}{{\text{DSC}}_{Ori}}\times 100(\%)$$

(7b) $$r_{tNLM/Ori}=\frac{DSC_{tNLM}}{{\text{DSC}}_{Ori}}\times 100(\%)$$

where DSC_*Ori*_, DSC_*DLSC*_ and DSC_*tNLM*_ are the similarity measurements between the group-level activation maps estimated from the original, DLSC-denoised and tNLM-denoised fMRI data with the HCP parcellation ROIs. As seen in Table 2, in terms of DSC and improvement ratios, the proposed DLSC-based denoising method has better capability to delineate the group-level activation detection results towards the ground truth, comparing with the results estimated from the original and the tNLM-denoised fMRI data.

### C. FUNCTIONAL CONNECTIVITY ANALYSIS: CEREBROCEREBELLAR CIRCUITS IN SOMATOMOTOR NETWORKS

**FIGURE 6.**
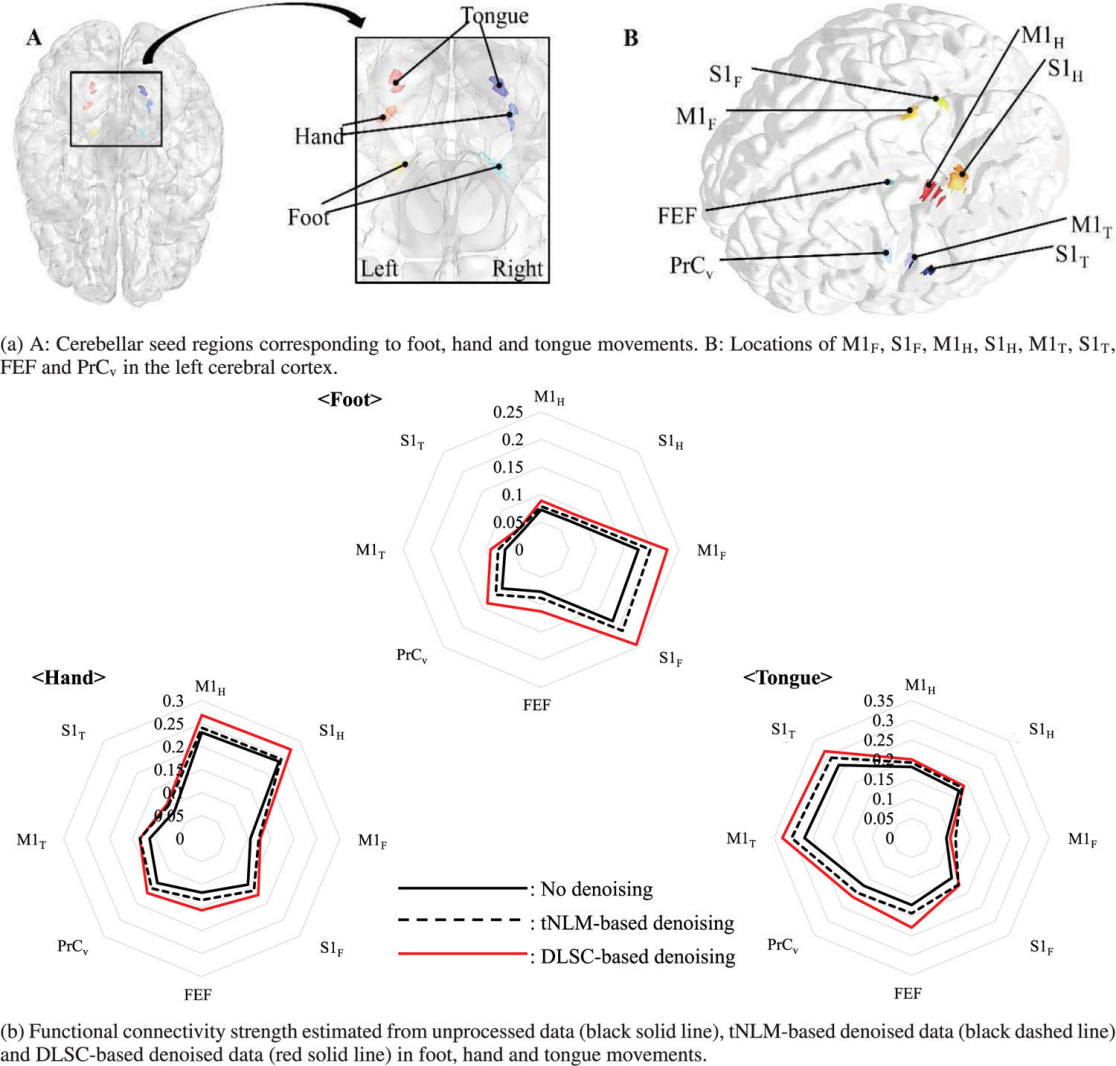
A: Cerebellar seed regions corresponding to foot, hand and tongue movements. B: Locations of M1_F_, S1_F_, M1_H_, S1_H_, M1_T_, S1_T_, FEF and PrC_v_ in the left cerebral cortex.

In this work, we use the cerebrocerebellar circuits involved in somatomotor networks as an example to analyze and validate the effectiveness of applying DLSC-based denoising for functional connectivity analysis. To this end, we consider the ground truth functional connectivity as reported in [2], [7] between three cerebellar seed regions of the foot, hand and tongue movements, and eight cerebral regions (M1_F_, S1_F_, M1_H_, S1_H_, M1_T_, S1_T_, FEF, PrC_v_) in each hemisphere as in Fig. 6, where F, H and T indicate foot, hand and tongue. According to [2], [7], the cerebral regions are supposed to be highly correlated with the cerebellar seed regions for the corresponding movement type. The frontal eye field (FEF) and the ventral precentral cortex (PrC_v_) at the caudal frontal cortex are part of the sensory-motor pathway. Each region consists of a single surface vertex (4 × 4mm) centering at the MNI coordinate tabulated in [2], [7], and the visualization is performed via BrainNet [35].

The group-averaged functional connectivity maps estimated from the original data (black solid line) and the denoised data by our proposed DLSC-based method (red solid line) and tNLM-based method (black dashed line) are visualized in Fig. 6b, for the foot, hand and tongue movements respectively. The functional connectivity is estimated by computing the Pearson’s correlation coefficient between the fMRI signals defined in the contralateral cerebral and cerebellar regions and averaging across the hemispheres. Fisher’s r-to-z transformation and its inverse transformation are further applied to normalize the distribution of the correlations. As shown in Fig. 6b, the proposed denoising method can strengthen the connectivity between the seed regions associated with each movement type and the corresponding M1 and S1 regions. The connectivities between M1_F_ and S1_F_ for foot movement, M1_H_ and S1_H_ for hand movement, M1_T_ and S1_T_ for tongue movement are increased through denoising. Moreover, the strengthening effect of the DLSC-based method is consistently more significant on the connectivities with high correlation (which are also neuroscientifically more meaningful), but less on the connectivities with low correlation estimated from the original signals (which are less meaningful and should not present). Thus, it can be concluded that stronger and more reliable functional connectivity patterns can be obtained through denoising by the proposed method, which cannot be obtained by the tNLM-based denoising method nor without denoising at all. In addition, the correlations at FEF and PrC_v_ become much more prominent after denoising by our method, showing that the sensory-motor pathway associated with visual cues could be revealed only through denoising. Such observation shows that the proposed DLSC-based denoising method can discover connectivity patterns which are previously indistinguishable due to the presence of noises disruption.

## IV. DISCUSSION AND CONCLUSION

In this work, we have studied the DLSC-based denoising method for the high-resolution brain activation localization and functional connectivity inference. Experimental results obtained from synthetic and real dataset show that the proposed method is capable of delineating more precise brain activation maps on both individual and group level, as well as providing more prominent functional connectivity patterns. Consistent and neuroscientifically meaningful functional localization and connectivity patterns which are previously disrupted by noises can be revealed by the proposed method. In-depth comparison with the latest non-local mean denoising method shows that the proposed method has the intrinsic advantage for high-resolution fMRI analysis due to its effectiveness in exploiting the sparse structure of the fMRI signals and its capability in avoiding smoothing the image. We envision that the proposed denoising method can be readily used on both fMRI and other functional neuroimaging methods (such as EEG) to reveal previously hidden brain architectures for neuroscience research and increase the capability for the brain-computer interface devices, thus offering a novel perspective in functional neuroimaging applications. The DLSC denoising code implemented in MATLAB, as well as all the supplemental figures can be found online at: https://xiangli-shaun.github.io/DLSC4fMRI/

## Supplementary Material

Supplemental Materials

## Figures and Tables

**TABLE 1.** Dice Similarity Coefficient (DSC) and the improvement ratio (*r*) based on the GLM activation detection experiments on synthetic fMRI data with additive Gaussian noise.

Movement	DSC_*Noised*_	DSC_*DLSC*_	DSC_*tNLM*_	*r*_*DLSC/Noised*_	*r*_*tNLM/Noised*_
*σ* = 0					
LF	-	0.8527	0.8499	-	-
LH	-	0.8841	0.8212	-	-
RF	-	0.8811	0.8534	-	-
RH	-	0.8431	0.8398	-	-
T	-	0.9038	0.9011	-	-
*σ* = 100					
LF	0.5599	0.6063	0.5546	108.2715%	99.0392%
LH	0.54	0.5842	0.5388	108.2%	99.7901%
RF	0.4299	0.5131	0.4255	119.3423%	98.9627%
RH	0.53	0.5914	0.5278	111.60%	99.60%
T	0.6699	0.7309	0.6664	109.0911%	99.4701%
*σ* = 200					
LF	0.2599	0.3490	0.2583	134.2551%	99.3730%
LH	0.2299	0.3279	0.2294	142.5975 %	99.7434%
RF	0.1599	0.2630	0.1596	164.4102%	99.7749%
RH	0.2299	0.3394	0.2287	147.6064 %	99.4738%
T	0.37	0.4830	0.3691	130.5594%	99.7810 %
*σ* = 300					
LF	0.1199	0.1968	0.12	164.0803%	100.0083%
LH	0.1099	0.2011	0.1098	182.8711%	99.8545%
RF	0.0799	0.1614	0.0799	201.7752 %	99.999%
RH	0.1099	0.1991	0.11	181.06%	100.0012%
T	0.2199	0.3268	0.21982	148.5885%	99.9227%

**TABLE 2.** Similarity between the group-level GLM activation maps from 3 different datasets and the ground truth, measured in Dice Similarity Coefficient (DSC), as well as the improvement ratios *r*_*DLSC/Ori*_ and *r_tNLM/Ori_* as defined in Eq. (7). In each movement type, the threshold *T_th_* for the t-values to define the activation maps is selected based on visual inspection. A full list of the comparisons using various levels of *T_th_* can be found in the Supplemental Materials, where the proposed method is shown to be capable of outperforming tNLM-based method in most cases.

Movement	DSC_*Ori*_	DSC_*DLSC*_	DSC_*tNLM*_	*r*_*DLSC/Ori*_	*r*_*tNLM/Ori*_
LF	0.3152	0.3278	0.3154	103.9975%	100.06345%
LH	0.4618	0.48	0.462	103.9411%	100.0433%
RF	0.3125	0.3163	0.3125	101.216%	100.01%
RH	0.2699	0.2762	0.2701	102.3341%	100.0741%
T	0.1841	0.1899	0.1842	103.1505%	100.0543%
