# Supplementary material for: Sparse Representation-Based Denoising for High-Resolution Brain Activation and Functional Connectivity Modeling: A Task fMRI Study
Source: IEEE Access. Author manuscript; Available in PMC 2022 May 6. (PMC9075697; doi:10.1109/access.2020.2971261)
Supplement: Supplemental Materials [file NIHMS1746845-supplement-Supplemental_Materials.pdf]

Dictionary Learning and Sparse Coding-based Denoising for High-Resolution Brain Activation and Functional Connectivity Modeling: A Task fMRI Study (Supplemental Materials)

Seongah Jeong[1*], Xiang Li[2*], Jiarui Yang[2], Quanzheng Li[2†], Vahid Tarokh[1]

Please note: All the supplemental figures, as well as the complete list of individual-level GLM results from all the 68 subjects can be found online at:

https://xiangli-shaun.github.io/DLSC4fMRI/

Supplemental Figure 1. Illustration of the SPM canonical hemodynamic response function (HRF) and six stimulus curves (visual cue, LF, LH, RF, RH, T) for motor task after convolution with the HRF function.

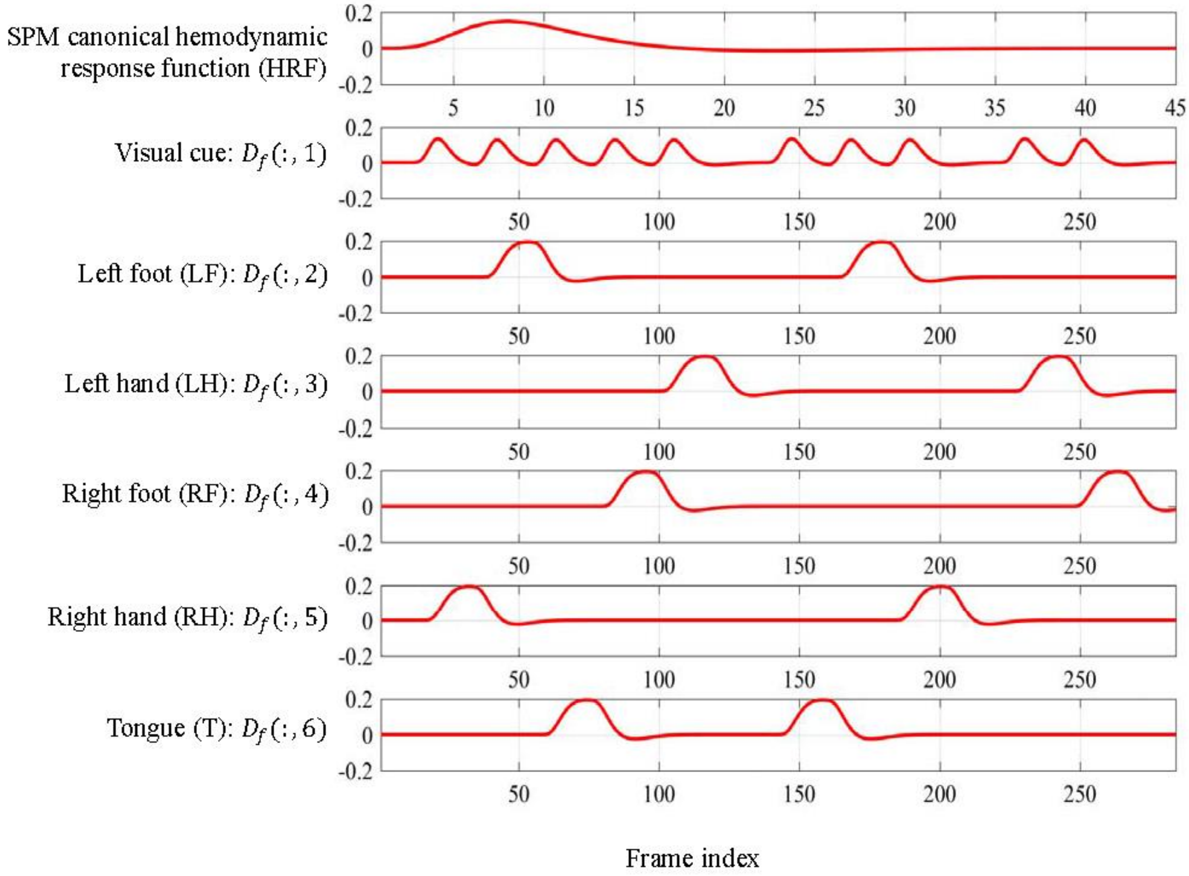

Supplemental Figure 2. Parameter tuning experiment showing the GLM-derived activation maps of DLSC-based denoised tfMRI data for LF, LH, RF, RH and T movements with the different K number of atoms, where λ=40 and C_{th}=0.1.

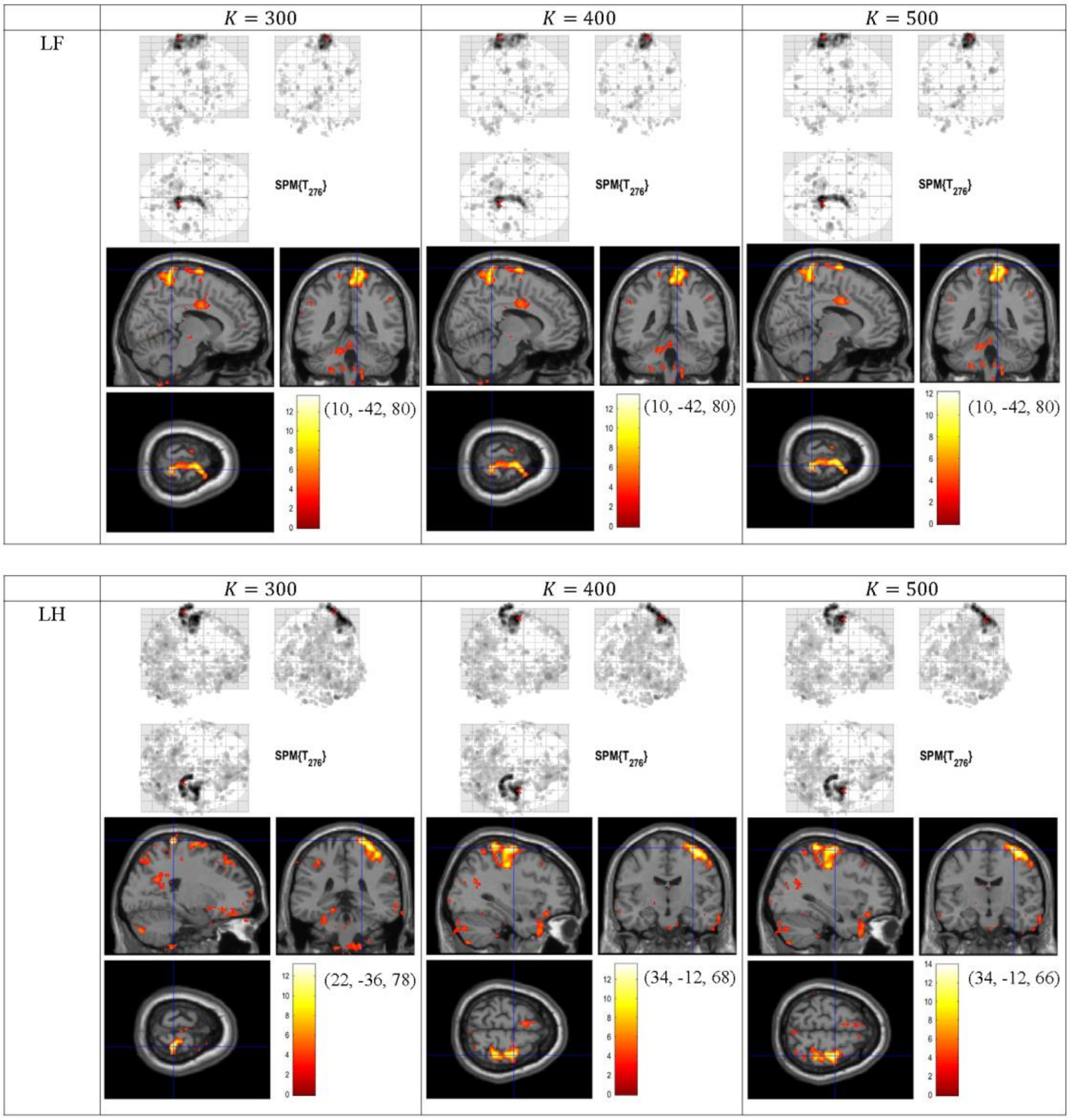

	$K = 300$	$K = 400$	$K = 500$
RF	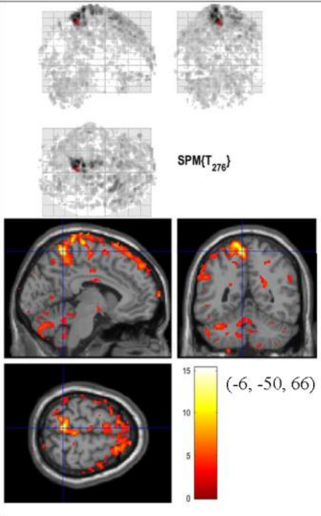 (-6, -50, 66)	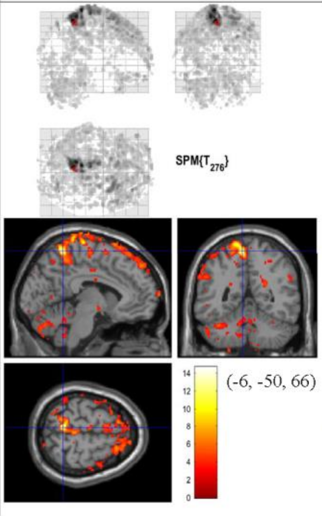 (-6, -50, 66)	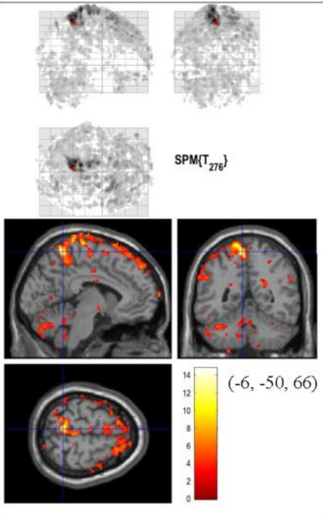 (-6, -50, 66)

	$K = 300$	$K = 400$	$K = 500$
RH	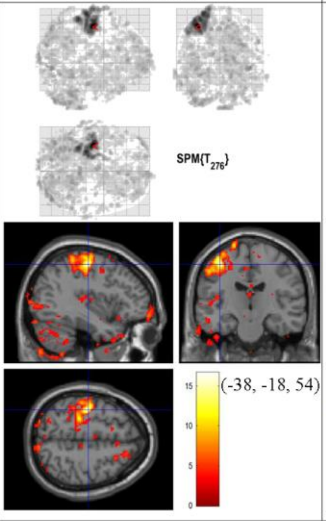 (-38, -18, 54)	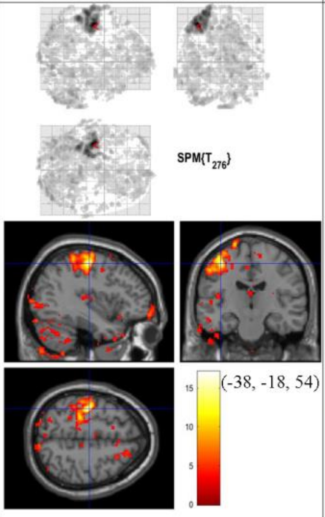 (-38, -18, 54)	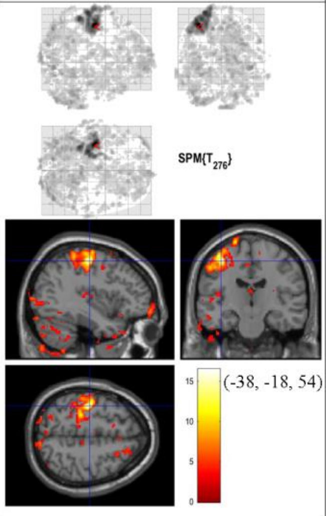 (-38, -18, 54)

	$K = 300$	$K = 400$	$K = 500$
T	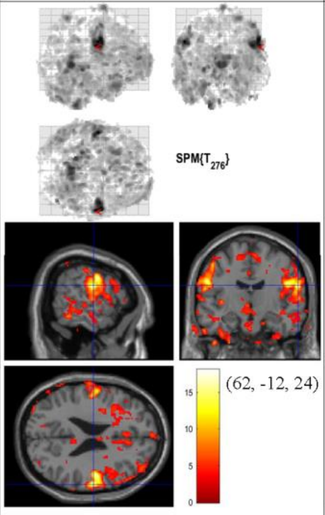 (62, -12, 24)	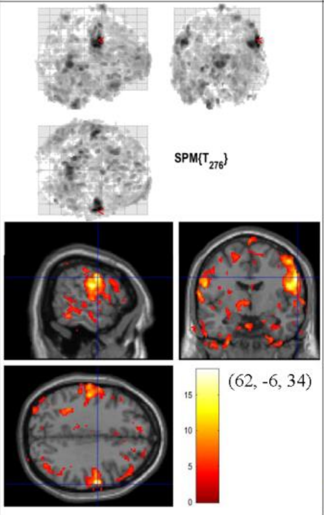 (62, -6, 34)	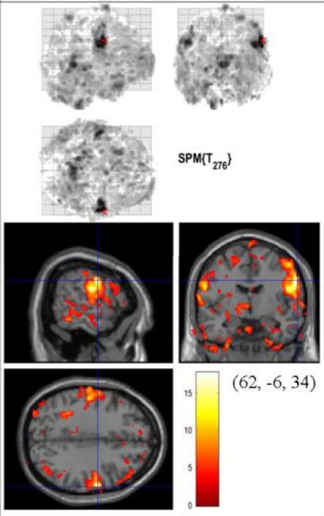 (62, -6, 34)

Supplemental Figure 3. Parameter tuning experiment showing the GLM-derived activation maps of DLSC-based denoised tfMRI data for LF, LH, RF, RH and T movements with the different sparsity constraint λ, where K=400 and C_{th}=0.1.

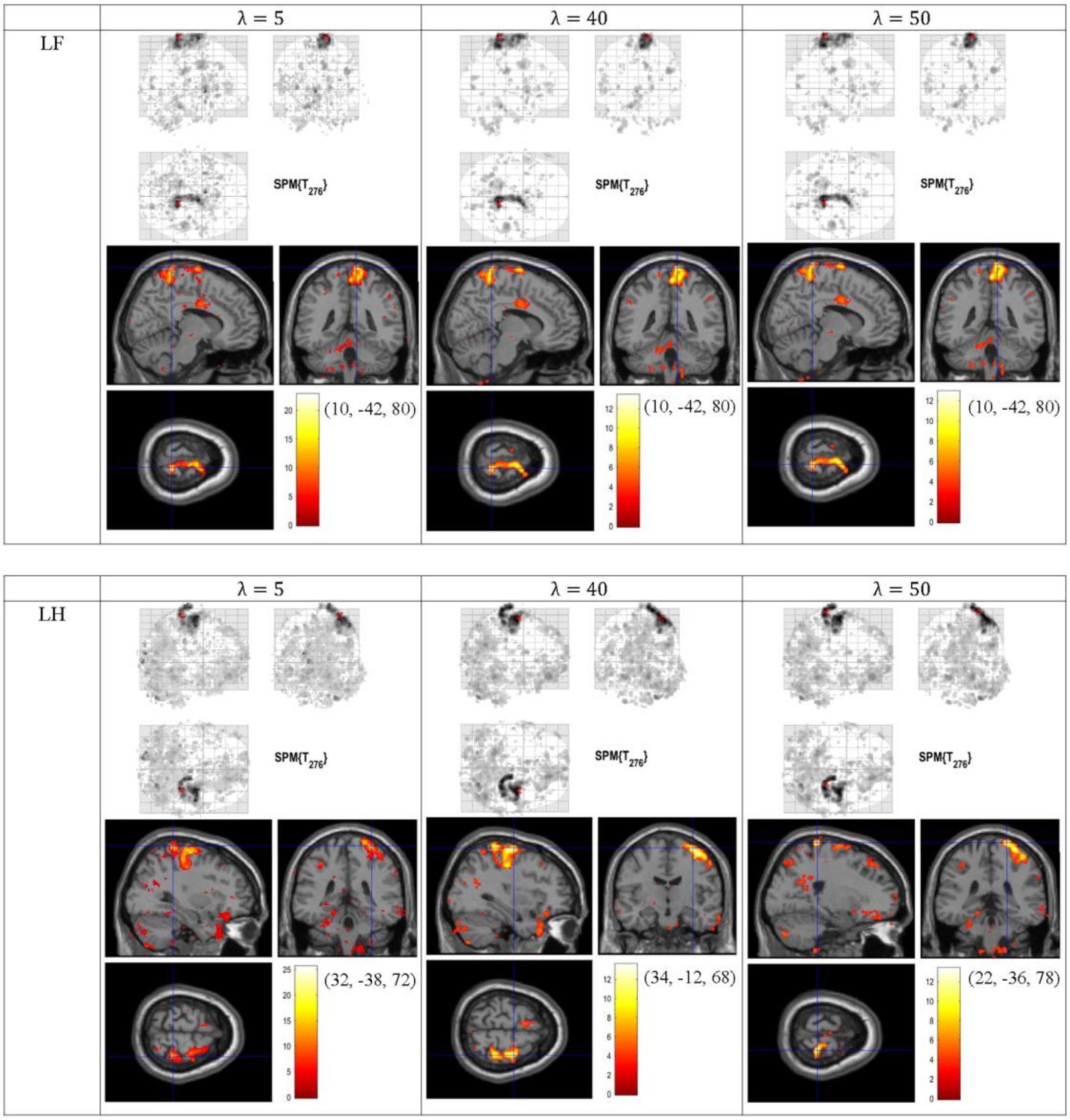

	$\lambda = 5$	$\lambda = 40$	$\lambda = 50$
RF	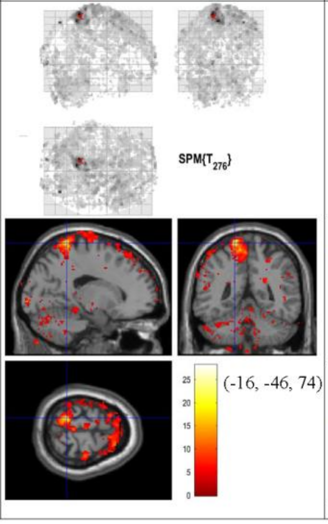	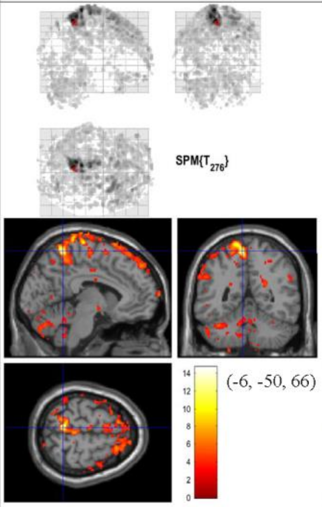	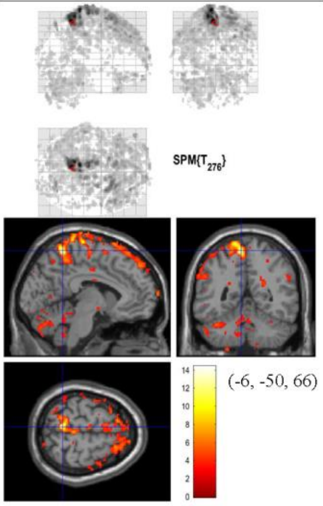

	$\lambda = 5$	$\lambda = 40$	$\lambda = 50$
RH	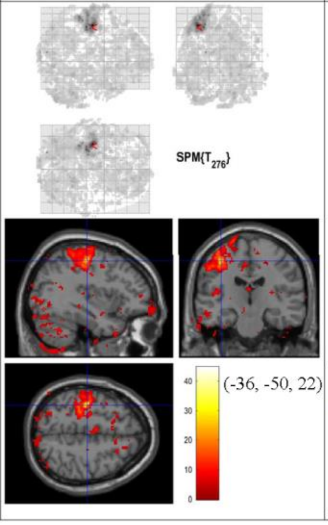	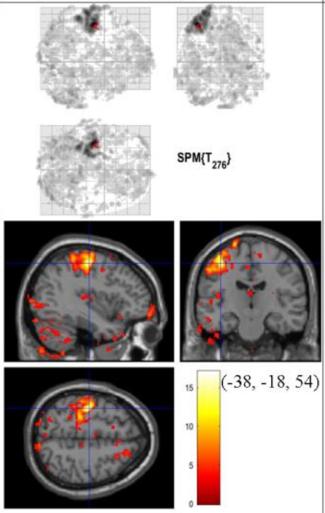	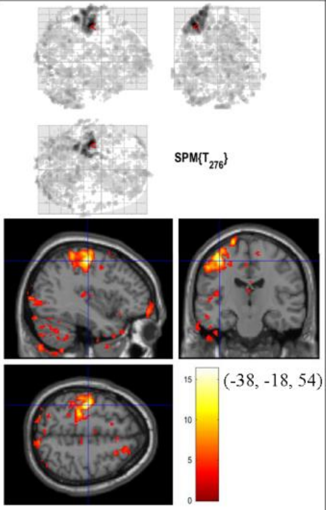

	$\lambda = 5$	$\lambda = 40$	$\lambda = 50$
T	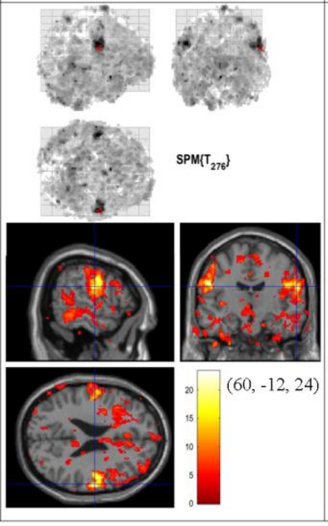	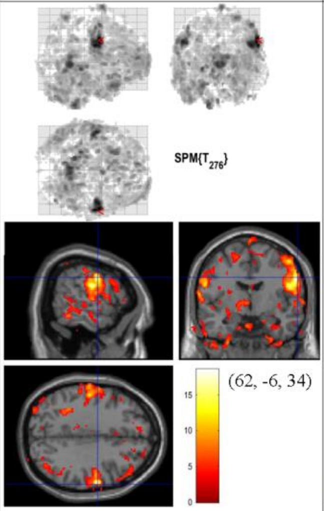	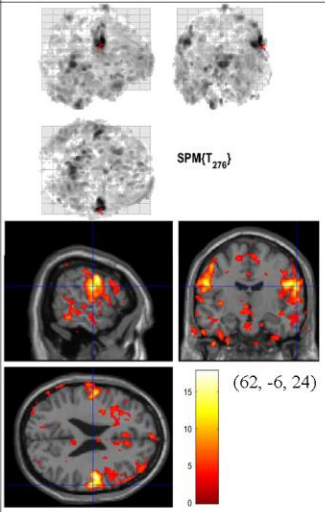

Supplemental Figure 4. Parameter tuning experiment showing the GLM-derived activation maps of DLSC-based denoised tfMRI data for LF, LH, RF, RH and T movements with the different signal-selection threshold value C_{th}, where K=400 and λ=40.

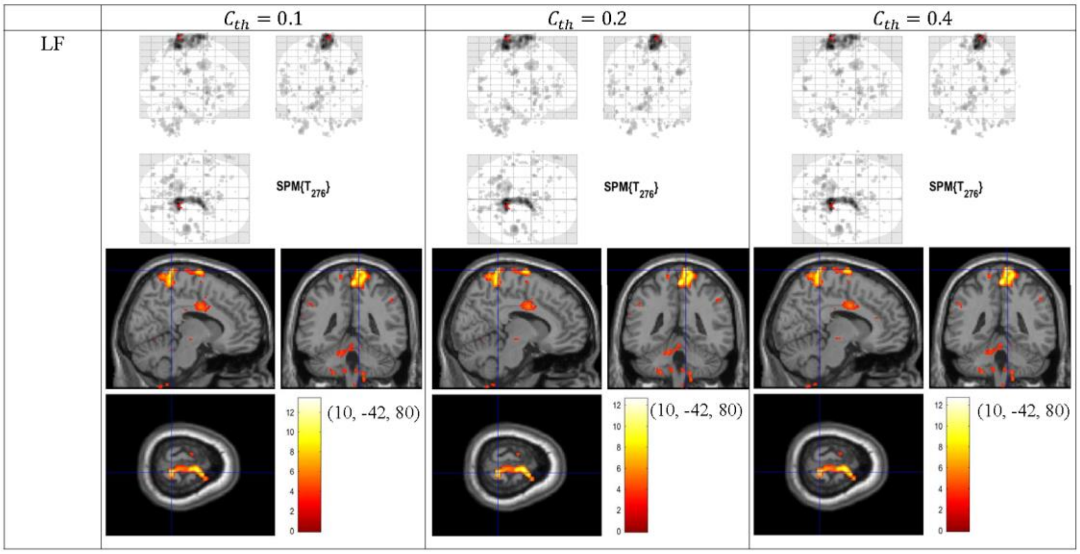

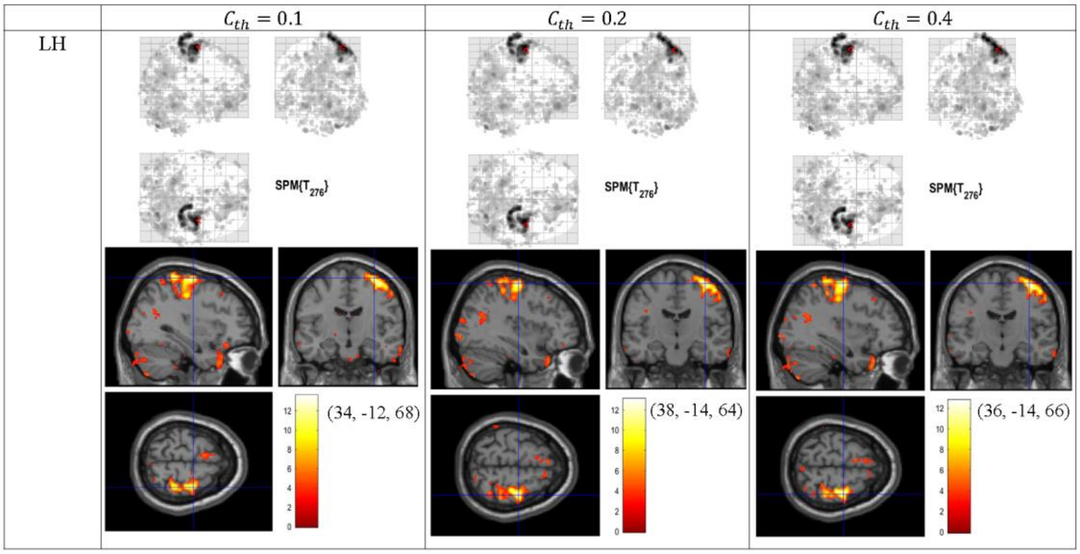

	$C_{th} = 0.1$	$C_{th} = 0.2$	$C_{th} = 0.4$
RF	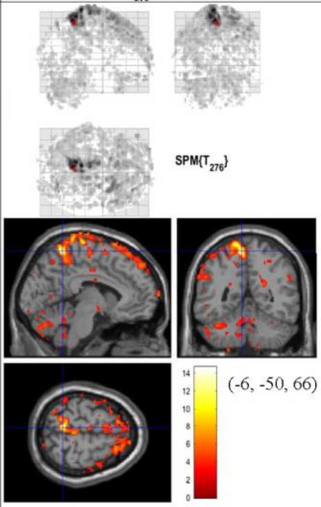	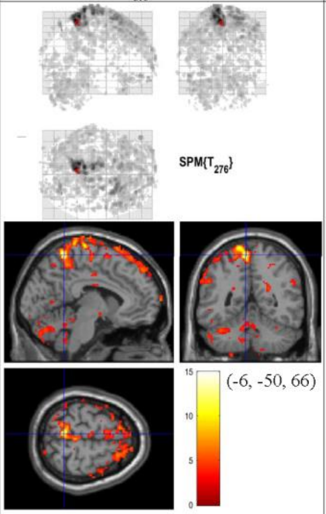	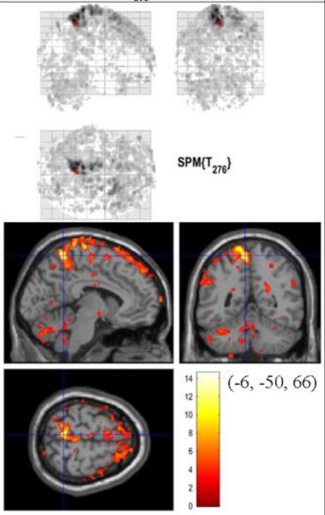

	$C_{th} = 0.1$	$C_{th} = 0.2$	$C_{th} = 0.4$
RH	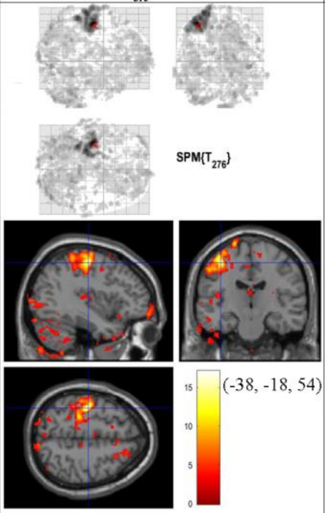	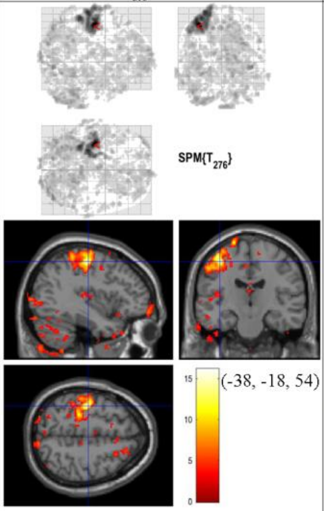	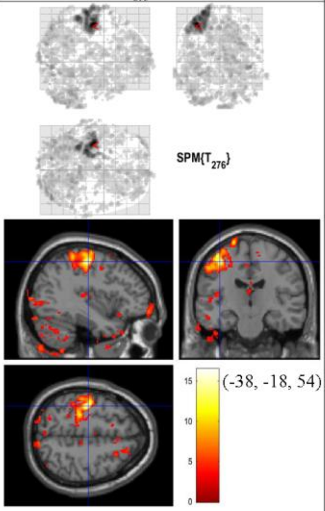

	$C_{th} = 0.1$	$C_{th} = 0.2$	$C_{th} = 0.4$
T	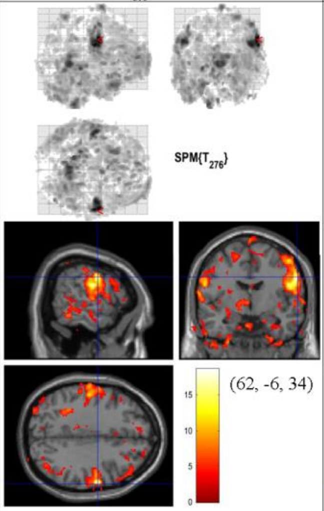	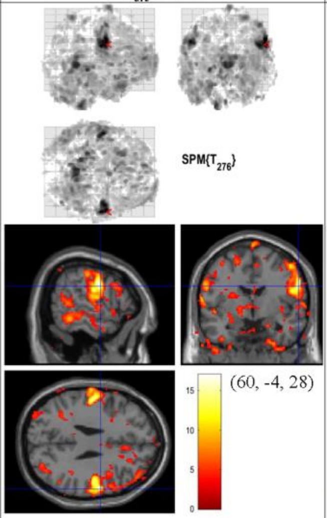	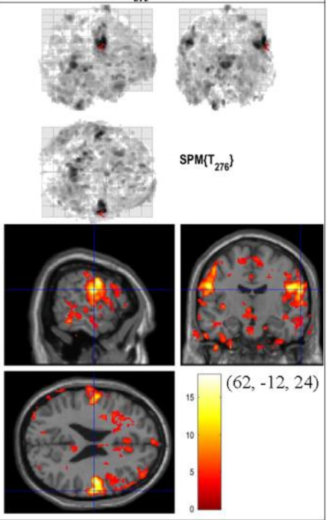

Supplemental Figure 5. The GLM-derived activation maps of original, noised and denoised synthetic tfMRI data by DLSC-based and tNLM-based method with σ=100.

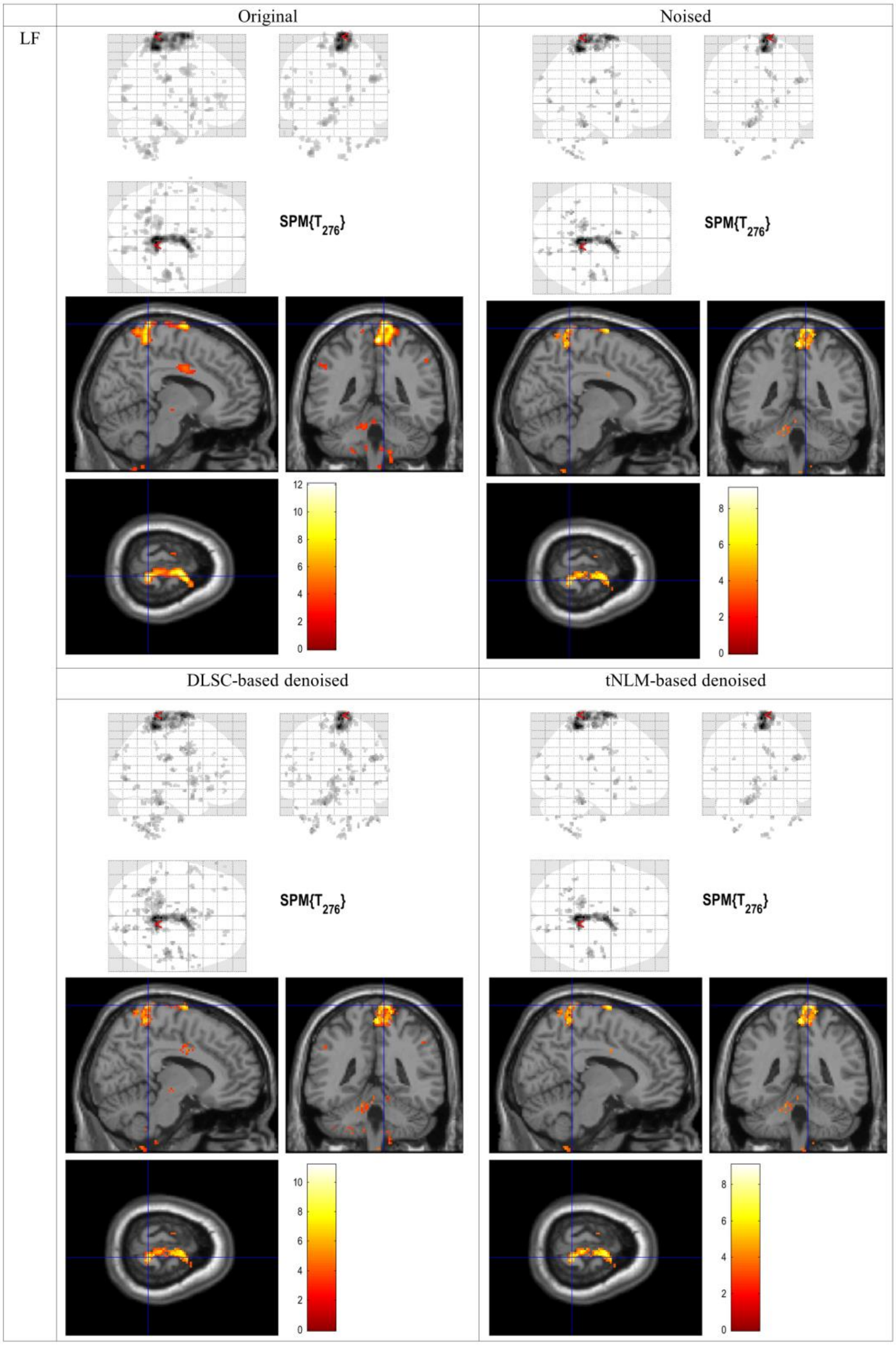

	Original	Noised
LH	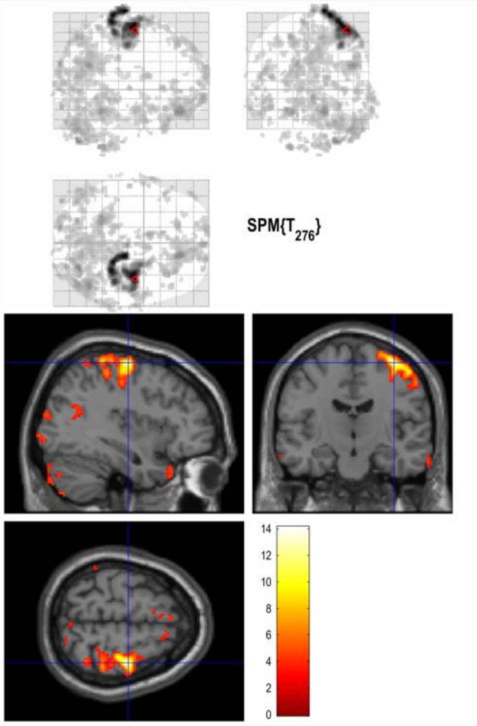	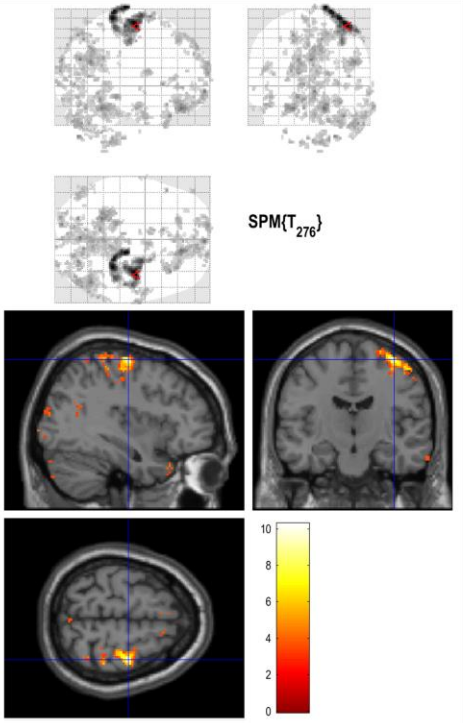
	DLSC-based denoised	tNLM-based denoised
	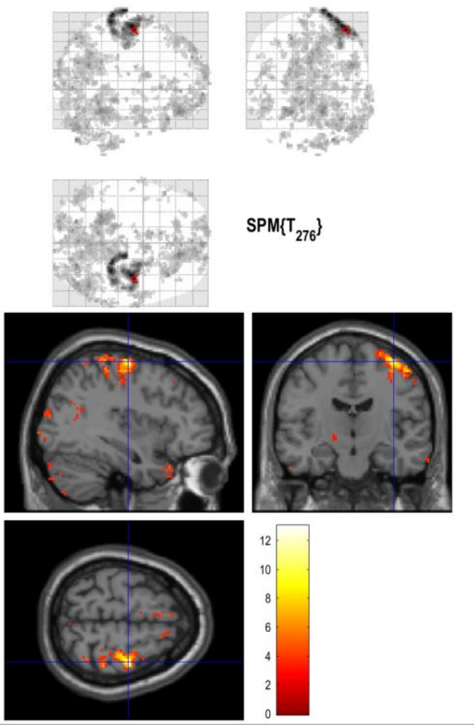	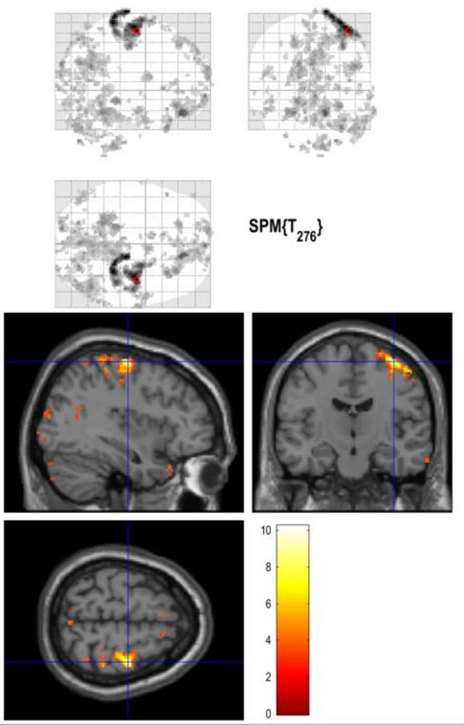

	Original	Noised
RF	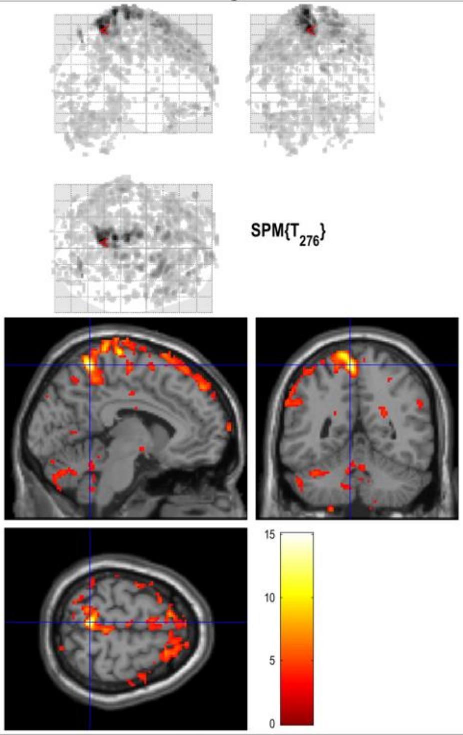	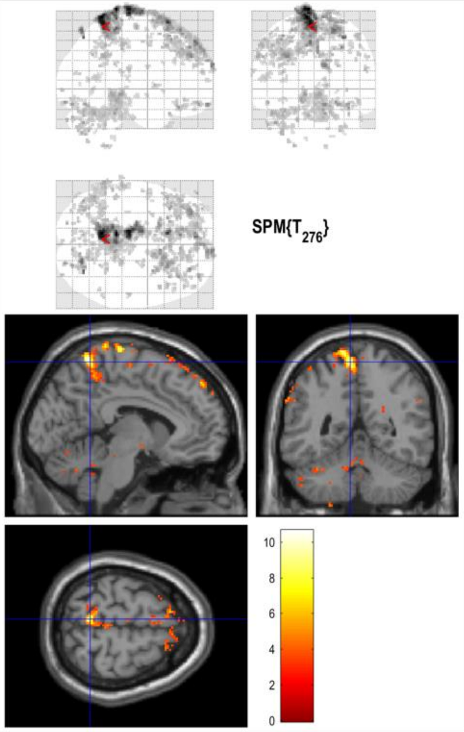
	DLSC-based denoised	tNLM-based denoised
	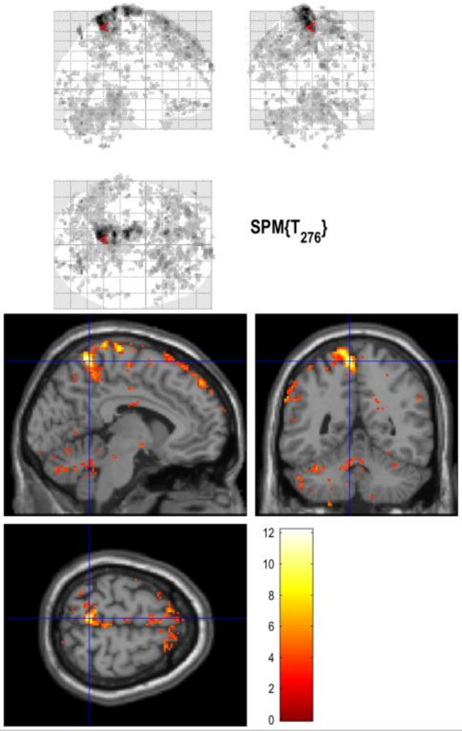	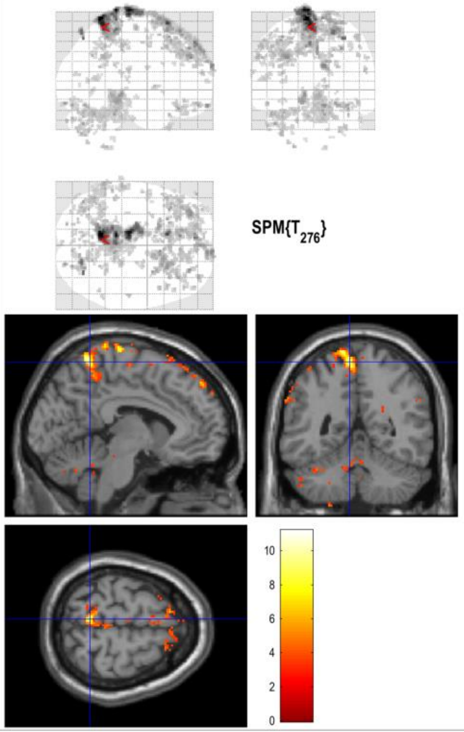

	Original	Noised
RH	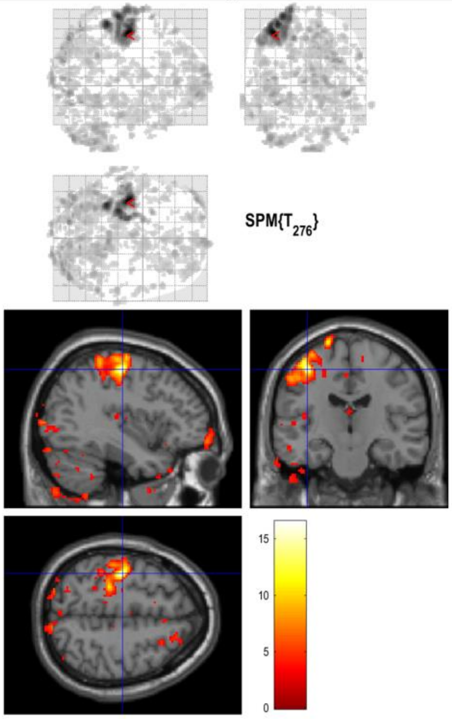	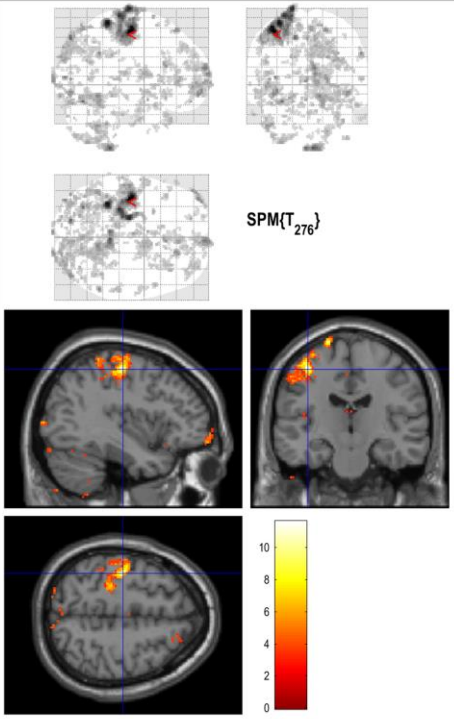
	DLSC-based denoised	tNLM-based denoised
	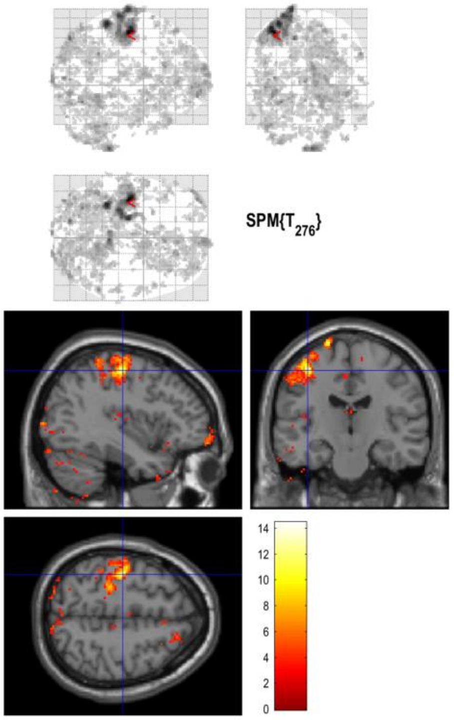	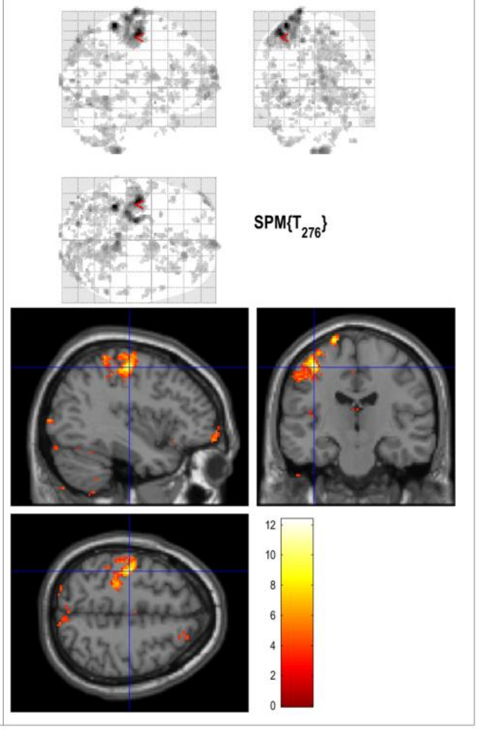

	Original	Noised
T	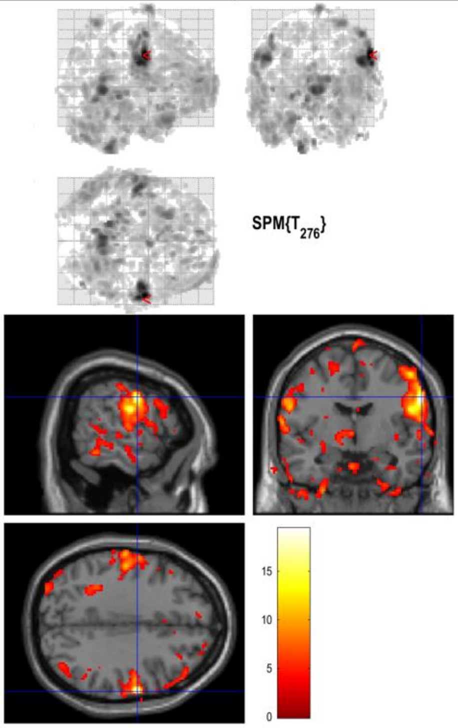	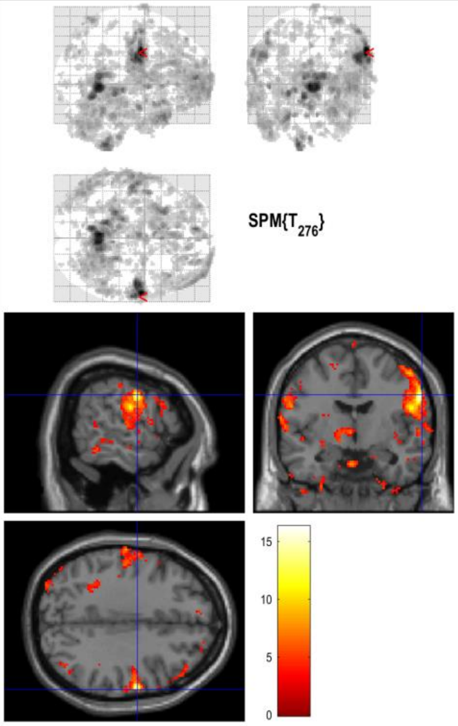
	DLSC-based denoised	tNLM-based denoised
	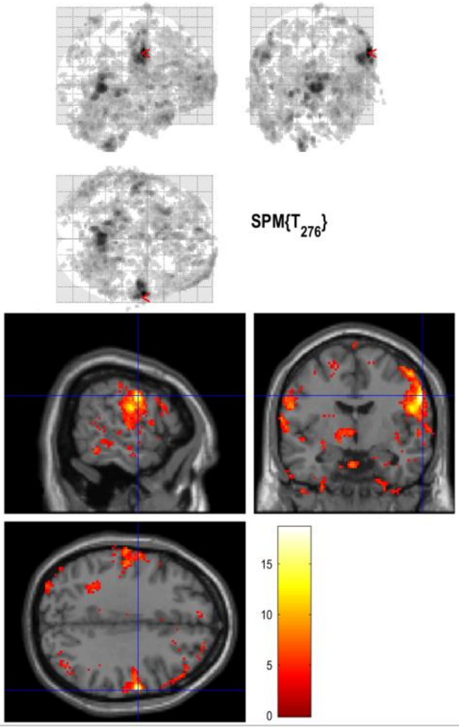	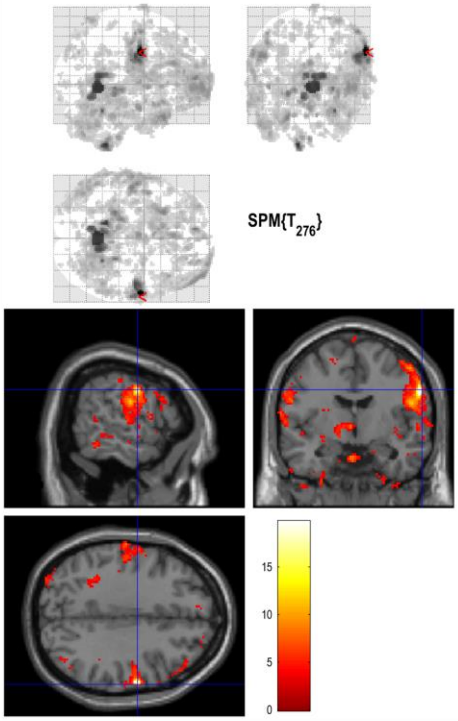

Supplemental Figure 6. The GLM-derived activation maps of original, noised and denoised synthetic tfMRI data by DLSC-based and tNLM-based method with σ=300.

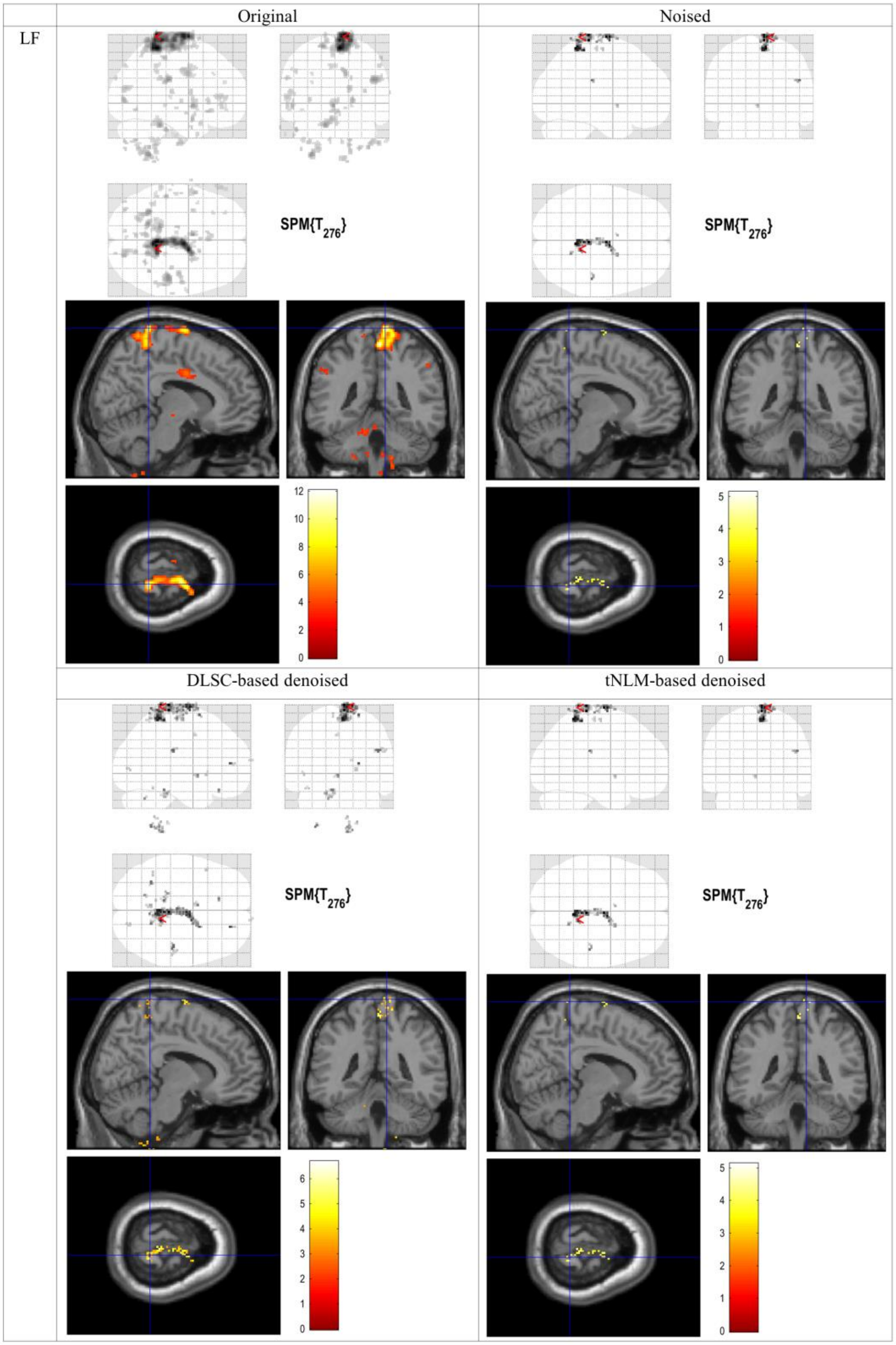

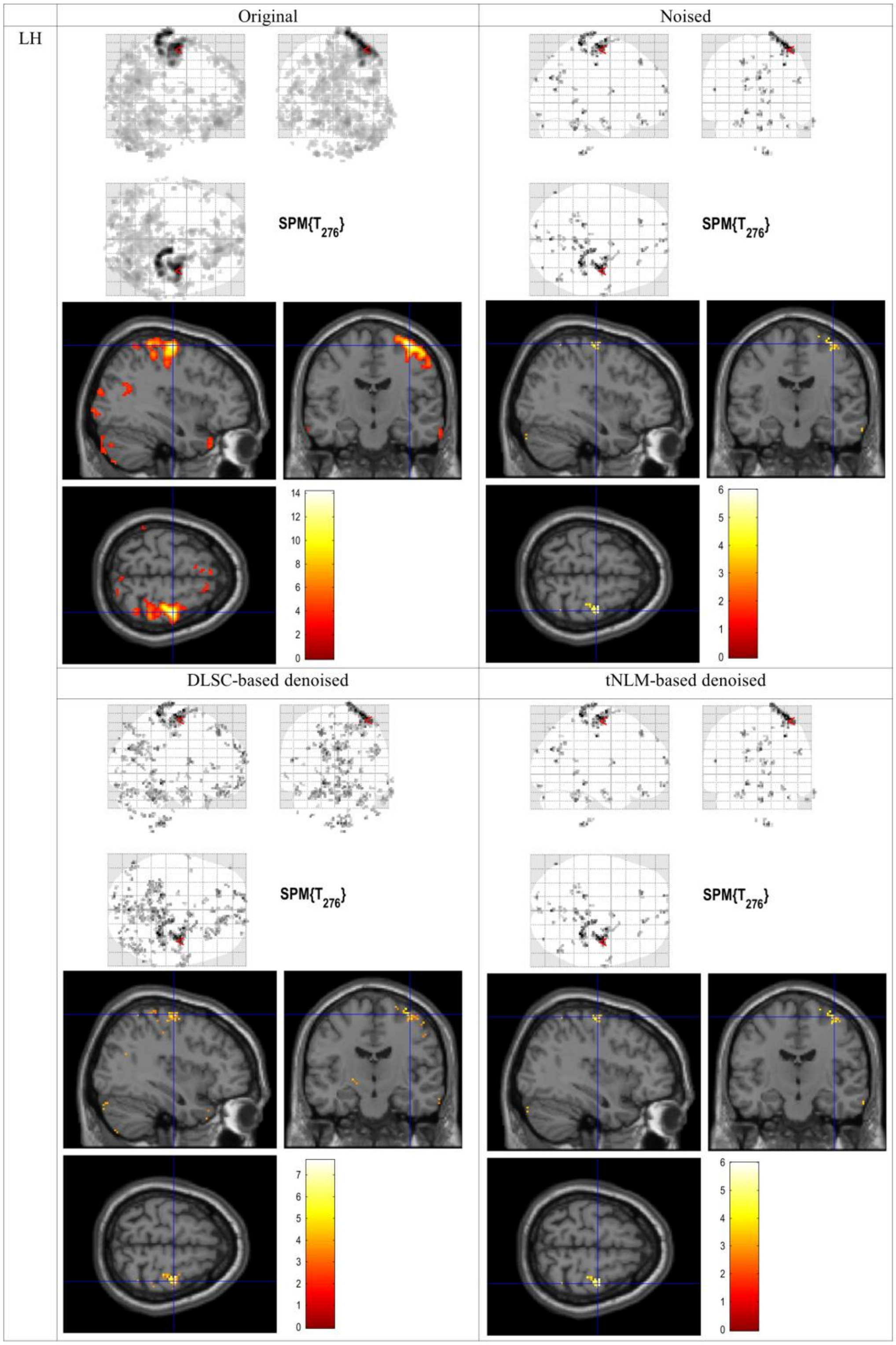
Original
Noised
LH
SPM{T276}
SPM{T276}
DLSC-based denoised
tNLM-based denoised
SPM{T276}
SPM{T276}

	Original	Noised
RF	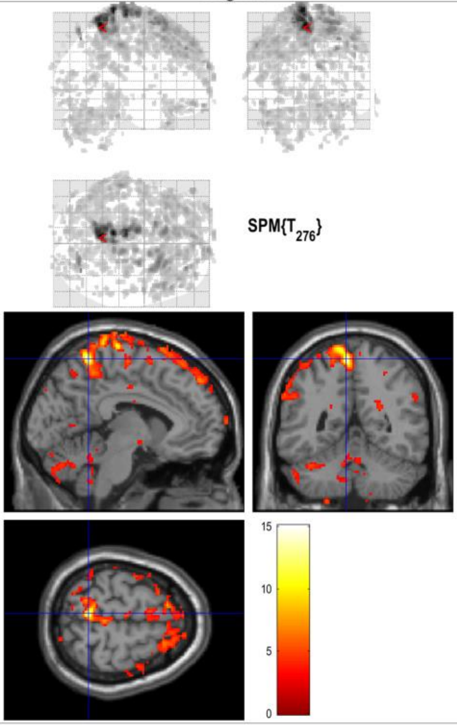	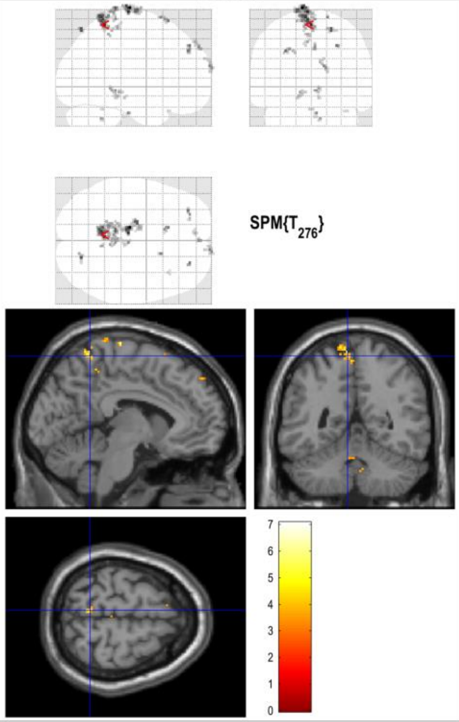
	DLSC-based denoised	tNLM-based denoised
	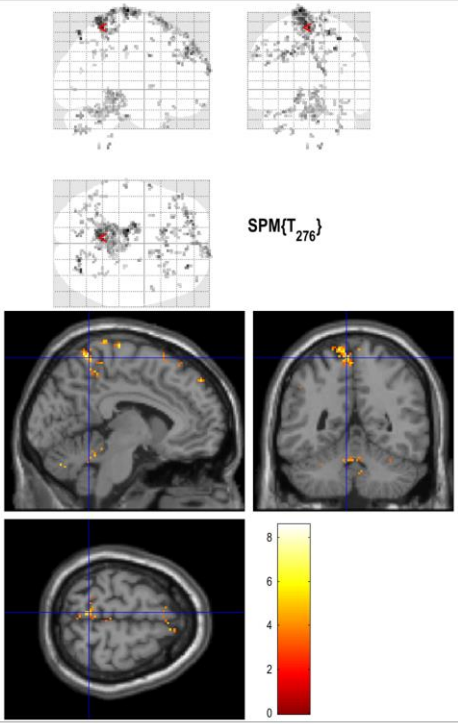	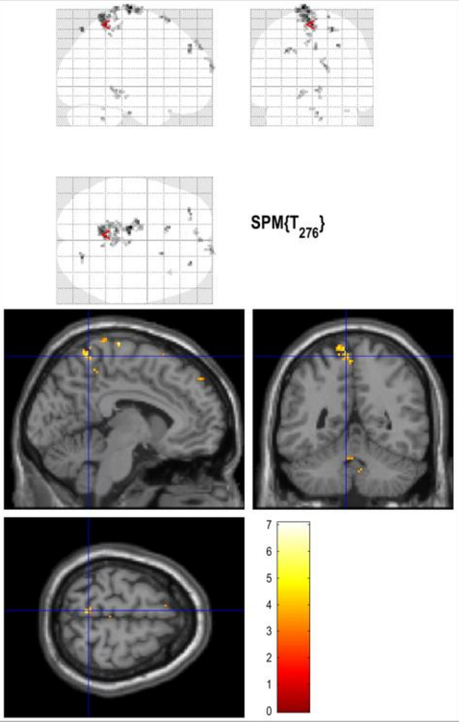

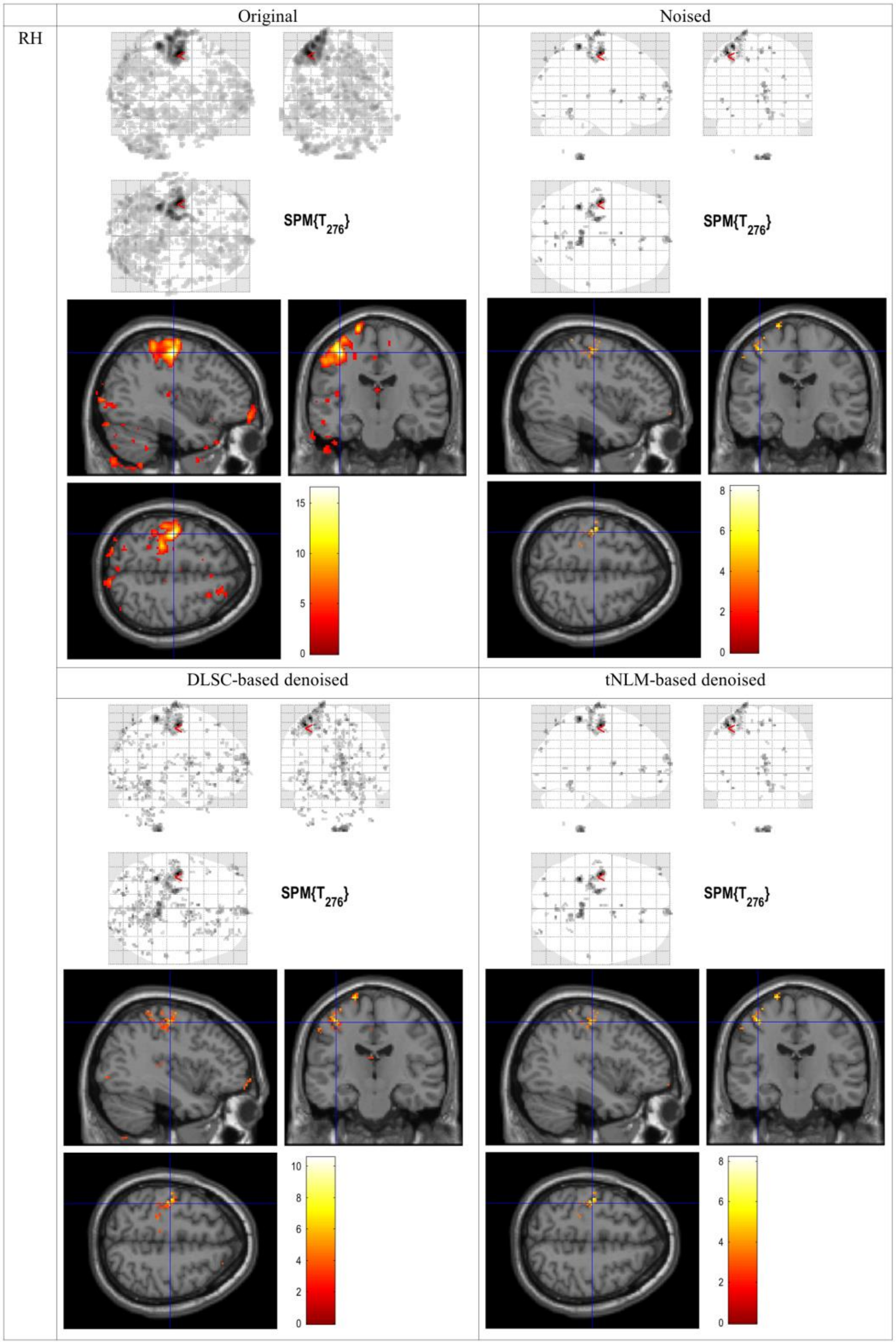
Original
Noised
RH
SPM{T276}
SPM{T276}
DLSC-based denoised
tNLM-based denoised
SPM{T276}
SPM{T276}

	Original	Noised
T	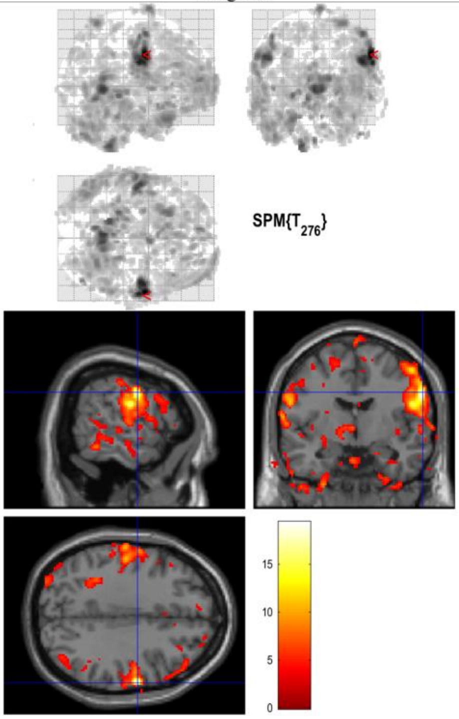	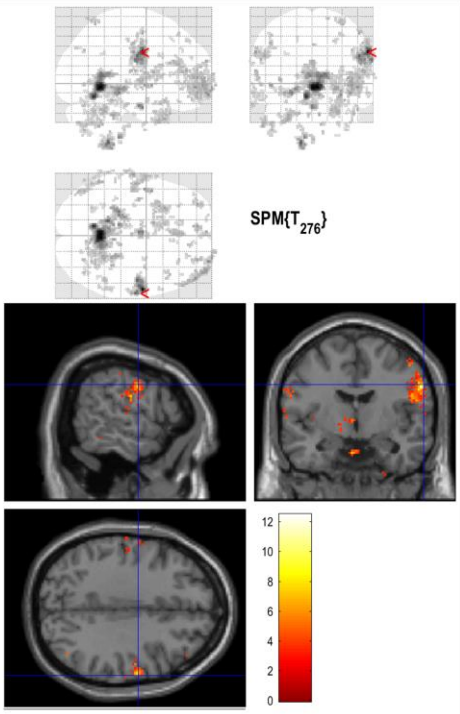
	DLSC-based denoised	tNLM-based denoised
	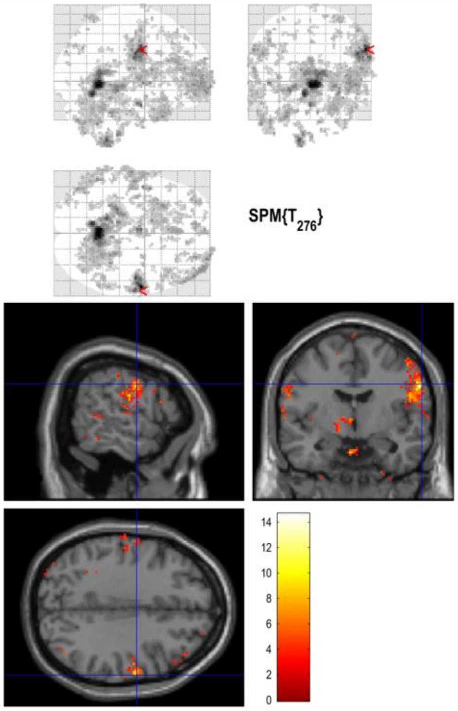	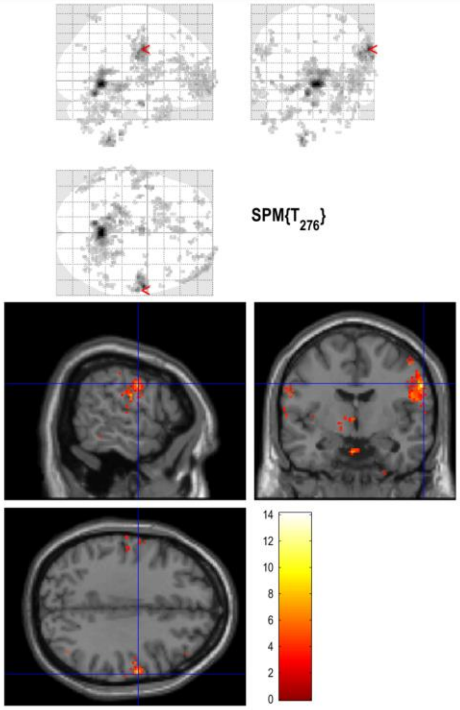

Supplemental Figure 7. The extracted ROIs obtained from the HCP parcellation for motor tasks reported in (Glasser et al., 2016).

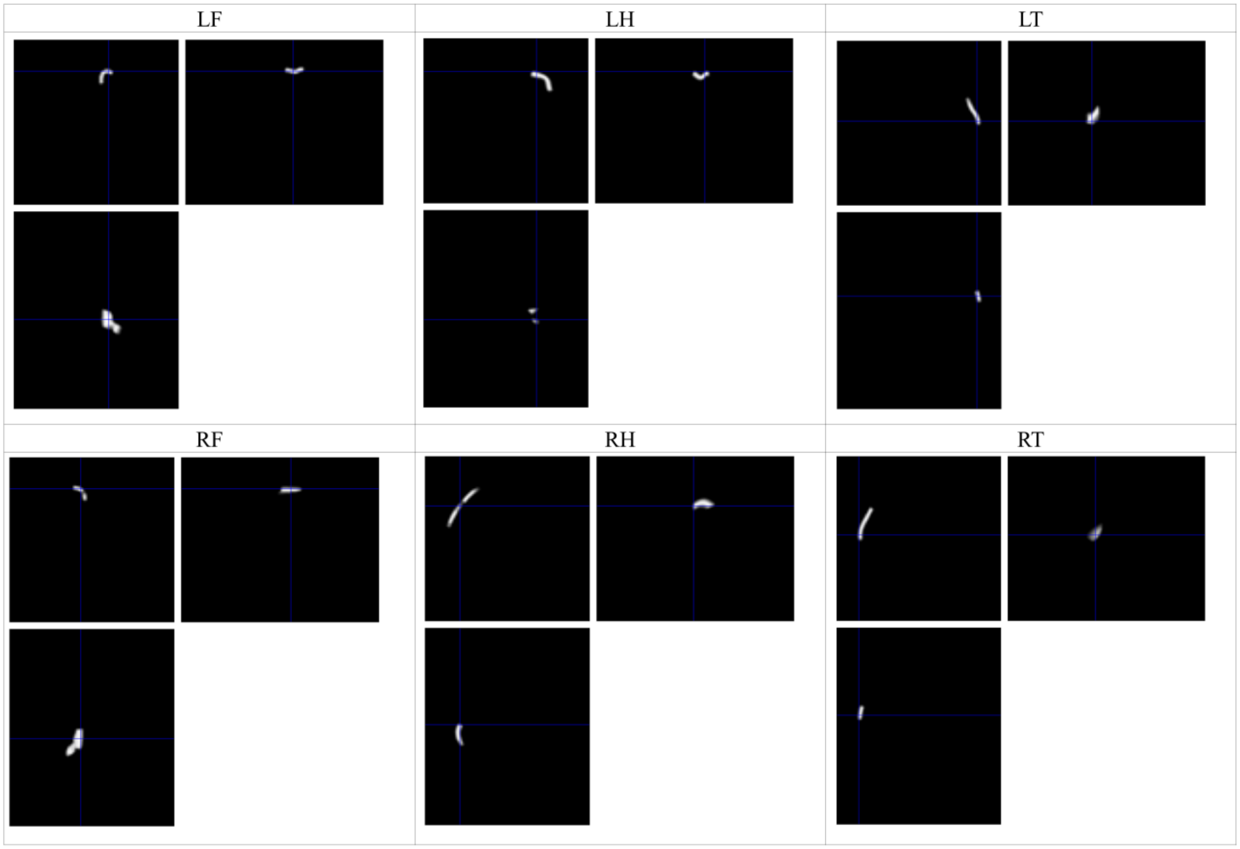

Supplemental Table 1. Comparisons of the denoising performance between the DLSC-based method and tNLM-based method using various level of *t*-value thresholds T_{th} (and corresponding *p*-values).

t -value	*p* -value	Average improvement rate of DLSC-based denoised image to the original image (r_{DLSC}) over 5 movements	Average improvement rate of tNLM-based denoised image to the original image (r_{tNLM}) over 5 movements	Ratio between r_{DLSC} and r_{tNLM} (r_{DLSC}/r_{tNLM})
6.14	2.5*10^-8	100.0267264	97.16640523	1.029437347
6.37	10^-8	101.4879271	98.7275795	1.027959235
6.54	5*10^-9	102.7135233	99.93650392	1.027787838
6.66	3*10^-9	103.6203144	100.8947448	1.02701399
6.93	10^-9	105.6280159	103.0589736	1.024927886
7.1	5*10^-10	106.8423506	104.4203629	1.023194591
8.04	10^-11	114.4936202	113.040435	1.012855446
8.59	10^-12	119.0913107	118.2581019	1.00704568
9.15	10^-13	124.1029184	123.9471896	1.001256412

References

Glasser, M.F., Coalson, T.S., Robinson, E.C., Hacker, C.D., Harwell, J., Yacoub, E., Ugurbil, K., Andersson, J., Beckmann, C.F., Jenkinson, M., 2016. A multi-modal parcellation of human cerebral cortex. Nature 536, 171-178.
